# Association Between Gut Microbiota and HIV Infection Risk: Insights from Mendelian Randomization and 16S rRNA Amplicon Sequencing

**DOI:** 10.3390/microorganisms14030667

**Published:** 2026-03-15

**Authors:** Jiali Chen, Tingting Yuan, Ji Pu, Ying Li, Han Zheng, Jing Yang, Jianguo Xu

**Affiliations:** 1 School of Medicine, Research Institute of Public Health, Nankai University, Tianjin 300071, China; chenjiali2525@163.com (J.C.); 15605533218@163.com (T.Y.); liying721622@163.com (Y.L.); 2 National Key Laboratory of Intelligent Tracking and Forecasting for Infectious Diseases, National Institute for Communicable Disease Control and Prevention, Chinese Center for Disease Control and Prevention, Beijing 102206, China; puji@icdc.cn (J.P.); zhenghan@icdc.cn (H.Z.); 3 Research Center for Reverse Microbial Etiology, Workstation of Academician, Shanxi Medical University, Taiyuan 030001, China

**Keywords:** HIV, mendelian randomization, gut microbiota, causality

## Abstract

Observational evidence links gut microbiota (GM) dysbiosis to HIV infection; however, the causal relationship between them has not been established. Mendelian randomization (MR) and 16S rRNA gene sequencing analyses were performed to identify gut microbial taxa associated with HIV infection risk. MR analysis results identified 18 gut microbial taxa associated with HIV infection (*p* values < 0.05), of which 16 taxa were detected in the 16S rRNA gene sequencing data. Following the exclusion of seven taxa with low relative abundance, eight taxa with potential relationships with HIV infection were detected in the 16S rRNA gene sequencing data. Four taxa (Clostridia class, Erysipelotrichales order, *Paraprevotella* genus, and *Parabacteroides distasonis* species) showed negative associations and four others (Proteobacteria phylum, *Coriobacteriaceae* family, *Subdoligranulum* genus, and *Bacteroides ovatus* species) showed positive associations with HIV infection risk. The eight taxa effectively distinguished between healthy controls (HCs) and people with HIV (PWH) (*p* values < 0.05). The area under the curve (AUC) values for the ROC curve analysis ranged from 0.62 to 0.87 for differentiating the HC and PWH groups. Furthermore, the effect of *Ruminococcus callidus* on HIV infection was partially mediated by hypoxanthine, exhibiting a mediated effect β of 0.17 (*p* = 0.042). These findings highlight the important role of the GM in HIV infection risk, facilitating future studies exploring better GM regulation strategies against HIV infection risk.

## 1. Introduction

Human immunodeficiency virus (HIV), the etiological agent of acquired immunodeficiency syndrome (AIDS), has posed a global public health challenge for several decades. The gut microbial community, a complex and dynamic ecosystem, is closely related to the occurrence and development of various diseases, including hypertension, type 1 diabetes and hepatitis B [1,2,3]. Recent research, utilizing 16S rRNA gene sequencing and metagenomic analysis has reported an association between HIV infection and gut microbiota (GM) dysbiosis [4,5]. These findings frequently note that HIV infection is accompanied by alterations in GM composition, such as increases in potentially pathogenic Proteobacteria and inflammatory genus *Prevotella*, and decreases in commensal bacteria (Bacteroidetes and Firmicutes) [6,7,8,9]. HIV-induced impairment of the intestinal mucosal barrier elicits inflammatory responses in both local and systemic areas in infected individuals. This increases intestinal mucosa permeability, in turn, promotes gut microbiota translocation, perpetuating immune activation and chronic inflammation (leading to increased levels of inflammatory cytokines such as TNF-α, IL-6, and IL-1β) [10,11]. In addition, the interaction between intestinal microbiota imbalance and metabolic abnormalities has been shown to be associated with a variety of pathological conditions. Dong et al. reported that plasma 25(OH)D was positively correlated with *Faecalibacterium*, Corprococcus_2 and Ruminococcaceae_NK4A214_groups, but negatively associated with Ruminococci_1, Eubacterium_eligens_group and uncultured_bacteria, in HIV infected individuals, suggesting a close relationship between blood metabolites and intestinal microbiota [12]. While numerous high-quality studies have been carried out, most are inherently correlational and cannot establish causality. The generalizability of microbiota data is often constrained by differences in sample size, control source, and confounders such as age, race, diet, medications, and personal behaviors, thereby producing inconsistent results across studies [13]. These inconsistencies, combined with the observational design of much existing work, preclude definitive causal inference regarding whether GM perturbations drive HIV susceptibility or progression, or vice versa. Meanwhile, the existence of interaction axes involving gut microbiota, blood metabolites or inflammatory cytokines, and HIV requires further investigation.

Observational studies on the association between HIV infection and GM are commonly limited by confounding, reverse causation, and population heterogeneity. These limitations constrain mechanistic understanding and identification of potential therapeutic targets. Mendelian randomization (MR) addresses these causal inference challenges by utilizing genetic variants as instrumental variables (IVs) [14]. MR validity relies on three core assumptions: (i) relevance, where IVs are strongly associated with the exposure (microbial features, inflammatory cytokines, blood metabolites); (ii) independence, where IVs are uncorrelated with confounders; and (iii) exclusion restriction, where IVs influence HIV infection risk only through the exposure [15]. Leveraging the random allocation of alleles at conception, MR effectively minimizes confounding bias and precludes reverse causation, thereby providing more reliable causal evidence than conventional observational studies [16,17]. MR has been successfully applied to explore causal relationships between GM and various diseases such as hepatitis B, liver cirrhosis and dementia [18,19,20]. To date, the causal relationship between HIV infection and GM has been explored in merely two MR investigations [21,22], both of which were limited to analyzing microbial taxa at the genus level or higher taxonomic ranks, leaving the relationship between HIV infection and GM at the species level unexplored.

The objective of this study is to integrate MR analysis with 16S rRNA amplicon sequencing to investigate the potential causal relationship between GM and HIV infection and elucidate the underlying mechanisms. We first employed a two-sample MR (TSMR) analysis framework utilizing large-scale genome-wide association study (GWAS) summary statistics to explore associations between GM and HIV infection. To corroborate these findings, we subsequently performed 16S rRNA gene sequencing to screen for microbial biomarkers associated with HIV infection. This process established a foundation for the exploration of potential microbial therapeutic targets. Finally, we explored the mediating roles of inflammatory cytokines and blood metabolites in the GM-HIV relationship.

## 2. Materials and Methods

### 2.1. Study Design

**Figure 1:**
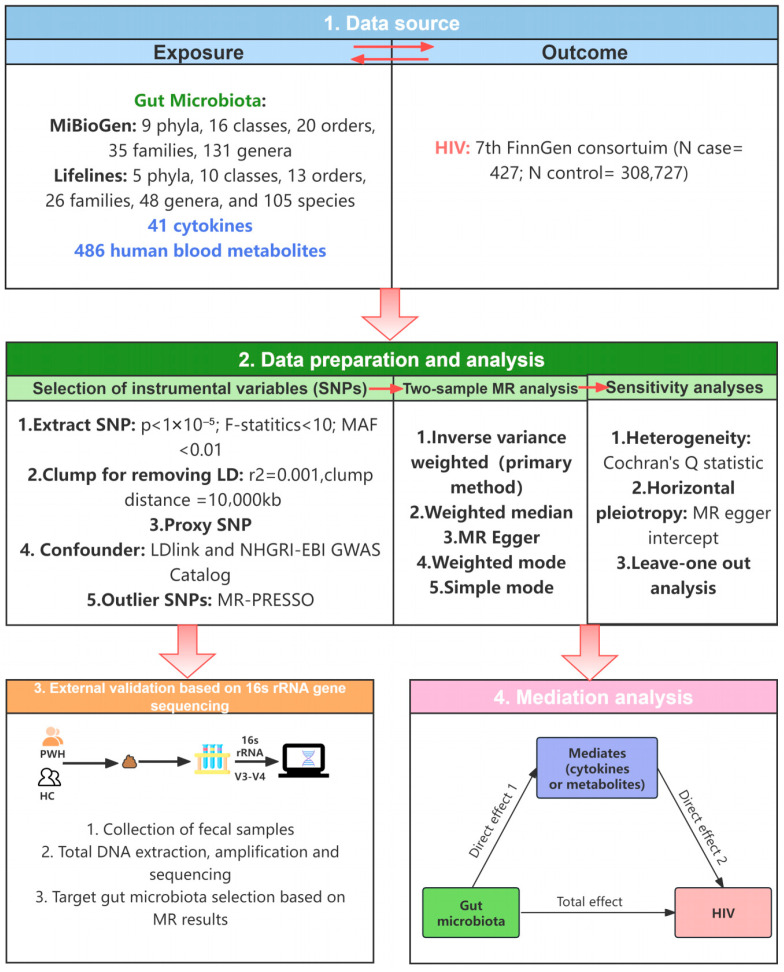
The flowchart of the study design.

The present study employed a multi-omics data integration strategy to explore the causal relationship and potential mechanism between GM and HIV infection (Figure 1). Firstly, GM was designated as the exposure factor, with gut microbiota-related single-nucleotide polymorphisms (SNPs) selected as instrumental variables (IVs). HIV infection was then designated as the outcome variable. MR analysis was adopted to estimate the potential causality between GM and HIV infection. Subsequently, we utilized previously collected 16S rRNA gene sequencing data from people with HIV (PWH) and healthy controls (HC) to validate the relative abundance differences in the GM suggested by the MR analysis results. Finally, potential mediators were explored, including 41 inflammatory cytokines and 486 human blood metabolites, that may mediate the effect of GM on HIV infection.

### 2.2. Data Sources

In this study, two GM datasets were incorporated as exposure data (Table S1). The first dataset originated from the large-scale GWAS meta-analysis by the MiBioGen consortium (https://mibiogen.gcc.rug.nl/, accessed on 1 June 2024). It encompassed 16S rRNA gene sequencing data from 18,340 participants (85% European ancestry) across 24 cohorts from the USA, Canada, Israel, South Korea, Germany, Denmark, The Netherlands, Belgium, Sweden, Finland, and the UK. The dataset comprised 211 gut microbial taxa (9 phyla, 16 classes, 20 orders, 35 families, 131 genera) [23]. The second dataset comprised metagenome sequencing data from 7738 participants from the Dutch Microbiome Project (DMP), covering 207 gut microbial taxa (5 phyla, 10 classes, 13 orders, 26 families, 48 genera, and 105 species) [24]. Furthermore, the GWAS summary data of 486 blood metabolites (309 known and 177 unknown metabolites) from 7824 adults in two European population studies [25] and 41 inflammatory cytokines from 8337 Finnish individuals [26] were included. GWAS summary statistics for HIV infection as the outcome were obtained from the 7th FinnGen consortium (https://www.finngen.fi/en, accessed on 1 June 2024), comprising 427 cases and 308,727 controls [27].

### 2.3. Instrumental Variable Selection

SNPs were initially selected as potential IVs using a genome-wide significance threshold (*p* < 5 × 10^−8^); however, this criterion yielded a limited number of genetic instruments (Table S2). Therefore, consistent with previous studies demonstrating that a less stringent threshold of *p* < 1 × 10^−5^ maintains data stability and accuracy, we adopted this threshold to ensure sufficient SNPs for robust instrument selection [20,21]. Subsequently, linkage disequilibrium (LD) clumping (r^2^ = 0.001 in the range of 10,000 kb) was executed to select SNPs with strong LD using the “clump_data” function from the “TwoSampleMR” R package based on the 1000 Genomes European reference panel [28]. The strength of IVs was evaluated by computing the F-statistic for each SNP according to Formula (1), with weak IVs (F < 10) excluded from subsequent analyses. SNPs exhibiting a minimum allele frequency (MAF) below 0.01 were removed from further analyses. SNPs significantly associated with traits potentially acting as confounders were identified using the NHGRI-EBI GWAS Catalog (https://www.ebi.ac.uk/gwas/, accessed on 1 June 2024) and excluded from all subsequent analyses. For exposure-related SNPs that were absent in the GWAS outcome data, proxy SNPs exhibiting high LD (r^2^ > 0.80) would be selected using the European 1000 Genomes data. The “harmonise_data” function was used to coordinate the direction of exposure-SNP and outcome-SNP alleles and to delete palindromic alleles. Finally, if the Mendelian Randomization Pleiotropy Residual Sum and Outlier (MR-PRESSO) global test indicates significant horizontal pleiotropy (*p* value < 0.05), the outlier SNPs were removed, and the remaining SNPs were reanalyzed using the MR analysis [29]. The same analysis steps were used to evaluate the causal relationship between metabolites or inflammatory cytokines as exposures and HIV infection.

(1) $$F=\frac{\left(N-k-1\right)}{k}\times \frac{R^{2}}{1-R^{2}}$$

### 2.4. Mendelian Randomization and Sensitivity Analyses

The present study employed five methods for causal effect estimation: inverse-variance weighted (IVW) [30], MR-Egger regression [31], weighted median estimator (WME) [32], simple mode (SM), and weighted mode (WM) [33]. The random-effects IVW approach, which aggregates Wald ratio estimations of various SNPs, was chosen as the primary MR analysis due to its higher statistical power compared to other approaches. To better interpret the causal relationships, the derived beta values were converted to odds ratios (ORs), with the corresponding 95% confidence intervals (CIs) also being calculated. Sensitivity analyses, incorporating leave-one-out analysis, heterogeneity testing and horizontal pleiotropy analysis, were performed to evaluate the robustness of the results. The “leave-one-out” function was used to reanalyse the results by eliminating IVs one by one, and the forest plots presented the effects of each SNP on the outcome. Cochran’s Q test, implemented via the “mr_heterogeneity” function, was utilized to evaluate possible bias in causal effect estimation arising from SNP measurement errors attributable to different analysis platforms, experimental conditions, and populations, with *p* value > 0.05 indicating negligible heterogeneity. In order to address the potential for bias resulting from horizontal pleiotropy, the MR-Egger regression method was employed. An intercept *p* value greater than 0.05 was indicative of the absence of horizontal pleiotropy [34]. Statistical power calculations were performed using the mRnd website [35]. The Benjamini–Hochberg (BH) method of false discovery rate (FDR) was applied to correct for multiple testing across all IVW analysis results. Given the exploratory nature of this study, which aimed to broadly investigate potential associations between GM and HIV infection, a relatively liberal significance threshold was adopted. Specifically, a corrected *p* value < 0.1 was considered suggestive of a potential causal relationship, while a corrected *p* value < 0.05 was interpreted as indicating a significant causal association [36]. A reverse MR analysis was performed to examine the potential causal effect of HIV infection (exposure) on the significant GM (outcome).

### 2.5. Mediation Analysis

The potential mediating roles of blood metabolites and inflammatory cytokines in the causal pathway from GM to HIV infection were evaluated using a two-step MR mediation analysis. The total causal effect (β3) of an exposure on an outcome comprises both the direct effect and the indirect effects through mediators. In this study, β3 was captured using a standard univariable MR analysis. In addition, in order to distinguish the direct effect and indirect effect, the direct effect (β1) of GM (exposure) on the mediators and the direct effect (β2) of the mediators on HIV infection (outcome) were estimated by MR analysis, respectively. The indirect effects were estimated using the product of coefficients method, with standard error (SE) and 95% CI derived by the Delta method [37]. The mediation proportion was calculated by dividing the indirect effect by the total effect (β1 × β2/β3) [22,38].

### 2.6. Target Gut Microbiota Association Analysis Based on 16S rRNA Gene Sequencing

The potential causal relationships between GM and HIV infection, as identified through univariable Mendelian randomization (UVMR) analysis, were externally validated using 16S rRNA gene sequencing data. Our team previously collected fecal samples from 58 HC and 114 PWH, from which total DNA was extracted [4]. Subsequently, the V3–V4 region of the 16S rRNA gene was amplified by PCR and sequenced by Majorbio Bio-Pharm Biotechnology Co., Ltd. (Shanghai, China). After quality filtering, sequences were processed using the DADA2 (Divisive Amplicon Denoising Algorithm, https://benjjneb.github.io/dada2/index.html, accessed on 1 January 2025) pipeline in QIIME2 to generate amplicon sequence variants (ASVs). Taxonomic assignment of ASVs was performed using the VSEARCH (version 2.22.1) classifier against the Human Gut Microbiome Analysis Database (HGMAD, DOI: 10.6084/m9.figshare.27281403, accessed on 21 April 2025). Sequencing depth averaged 87,638 ± 9974 reads per sample in the HC group, and 94,585 ± 38,862 reads per sample in the PWH group. Relative abundances were calculated using the “amplicon” package in R4.3.1. Target microbial taxa with an average relative abundance of less than 0.1% were excluded from the subsequent analysis [39]. The relative abundance of each target microbial taxon between the HC and PWH groups was selected and compared using the Mann–Whitney U test (*α* = 0.05). In addition, the predictive value of each target microbial taxon as a biomarker for distinguishing the HC and PWH groups was evaluated by constructing receiver operating characteristic (ROC) curves and calculating the area under the curve (AUC) values.

## 3. Results

### 3.1. Causal Effects of Gut Microbiota on HIV

**Figure 2:**
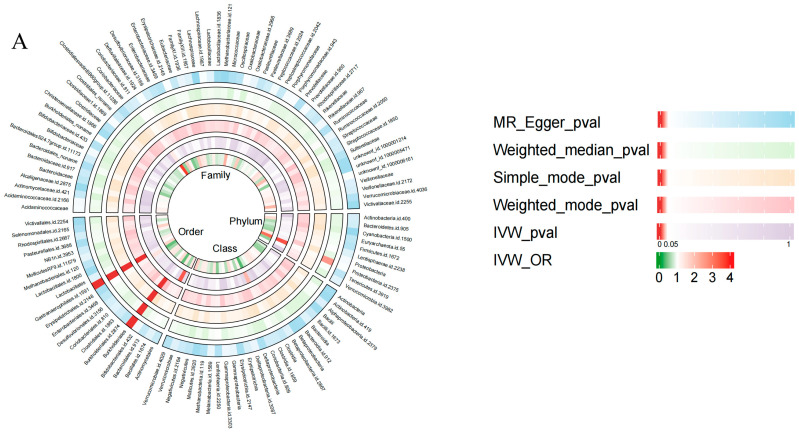

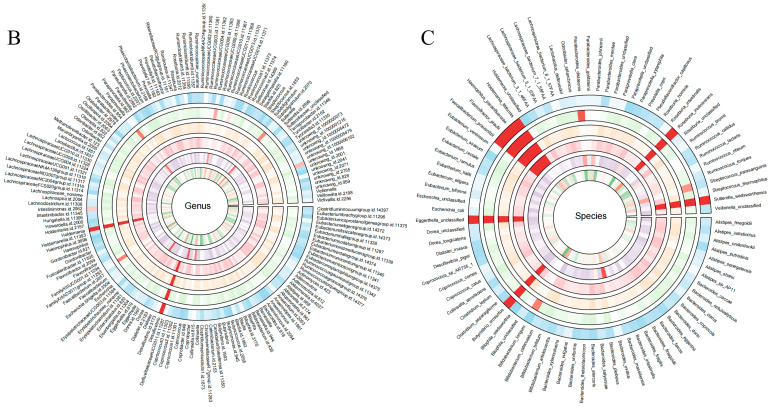
Circular heatmaps displaying Mendelian randomization results for gut microbiota (exposure) and HIV infection (outcome). The circular heatmaps display, from outer to inner rings, the *p* values from MR-Egger, weighted median, simple mode, and weighted mode analyses, followed by the *p* values and odds ratio (OR) from the IVW analysis. (**A**) Phylum, class, order, and family levels; (**B**) genus level; (**C**) species level.

**Figure 3:**
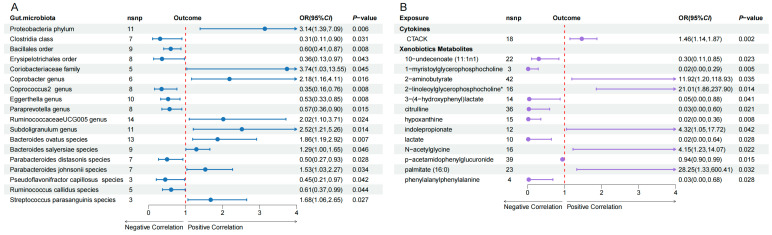
The forest plot shows the causal relationships (**A**) between gut microbiota and HIV infection and (**B**) between cytokines or metabolites and HIV infection. The asterisk (*) denotes that the compound was not confirmed with a pure standard.

Based on the IV selection criterion of *p* < 1 × 10^−5^, 3804 SNPs were associated with GM at six taxon levels for further analyses. MR analysis of these selected IVs suggested 18 bacterial taxa as potential HIV-influencing factors: one phylum, one class, two orders, one family, six genera, and seven species (Figure 2, Table S3). As shown in Figure 3A, IVW analysis results indicated that nine protective factors against HIV infection at different taxonomic levels, including Clostridia class (OR = 0.31, 95% CI: 0.107–0.899, *p* = 0.031), Bacillales order (OR = 0.597, 95% CI: 0.408–0.874, *p* = 0.008), Erysipelotrichales order (OR = 0.361, 95% CI: 0.135–0.967, *p* = 0.043), *Eggerthella* genus (OR = 0.526, 95% CI: 0.326–0.847, *p* = 0.008), Coprococcus2 genus (OR = 0.349, 95% CI: 0.16–0.763, *p* = 0.008), *Paraprevotella* genus (OR = 0.566, 95% CI: 0.357–0.896, *p* = 0.015), *Parabacteroides distasonis* species (OR = 0.497, 95% CI: 0.267–0.926, *p* = 0.028), *Pseudoflavonifractor capillosus* species (OR = 0.448, 95% CI: 0.207–0.971, *p* = 0.042) and *Ruminococcus callidus* species (OR = 0.607, 95% CI: 0.374–0.986, *p* = 0.044). Conversely, nine taxa, including Proteobacteria phylum (OR = 3.138, 95% CI: 1.39–7.085, *p* = 0.006), *Coriobacteriaceae* family (OR = 3.737, 95% CI: 1.031–13.545, *p* = 0.045), *Subdoligranulum* genus (OR = 2.518, 95% CI: 1.205–5.26, *p* = 0.014), *Coprobacter* genus (OR = 2.184, 95% CI: 1.159–4.113, *p* = 0.016), *RuminococcaceaeUCG005* genus (OR = 2.016, 95% CI: 1.096–3.709, *p* = 0.024), *Bacteroides ovatus* species (OR = 1.862, 95% CI: 1.189–2.916, *p* = 0.007), *Streptococcus parasanguinis* species (OR = 1.676, 95% CI: 1.061–2.648, *p* = 0.027), *Parabacteroides johnsonii* species (OR = 1.531, 95% CI: 1.033–2.268, *p* = 0.034), and *Bacteroides salyersiae* species (OR = 1.289, 95% CI: 1.004–1.654, *p* = 0.046), were identified as HIV infection risk factors. After FDR correction, Proteobacteria phylum showed a suggestive association with HIV infection (*q* = 0.059), while all other gut microbiota were no longer statistically significant (*q* values > 0.1). Table S3 outlines the result of statistical power for IVM analysis and four additional methods (weighted median, MR-Egger, weighted mode, and simple mode) that reinforced the findings by the IVW method. The relationships between the 18 gut microbial taxa and HIV infection proved the robustness, with no evidence of horizontal pleiotropy (*p* values > 0.05), heterogeneity (*p* values > 0.05), and outliers (global test *p* values > 0.05) (Table S4). Furthermore, leave-one-out analysis of the 18 gut microbial taxa (Figure S1) confirmed that no single SNP dominated the causal associations with HIV infection.

### 3.2. Causal Effect of Cytokines and Metabolites on HIV

A total of 745 SNPs strongly associated with inflammatory cytokines and 8837 SNPs significantly associated with human blood metabolites were included in the UVMR analysis. The IVW analysis results indicated that IL-17 and cutaneous T-cell attracting chemokine (CTACK) might be potential influencing factors for HIV infection. The MR-PRESSO analysis for IL-17 suggested the presence of outlier SNPs (global test *p* = 0.008). After removing these outliers and repeating the MR analysis, the estimated causal effect of IL-17 on HIV infection was not significant (OR = 0.81, 95% CI: 0.51–1.29, *p* = 0.370), resulting in its exclusion from subsequent analyses. As shown in Figure 3B and Table S5, CTACK (OR = 1.46, 95% CI: 1.14–1.87, *p* = 0.002) was identified as a risk factor for HIV infection. In the UVMR results of human blood metabolites, we identified thirteen metabolites significantly associated with HIV infection (*p* values < 0.05). Eight metabolites showed negative associations, including 1-myristoylglycerophosphocholine, lactate, hypoxanthine, phenylalanylphenylalanine, citrulline, 3-(4-hydroxyphenyl) lactate, 10-undecenoate (11:1n1) and p-acetamidophenylglucuronide. Five metabolites demonstrated positive associations, including N-acetylglycine, indolepropionate, 2-aminobutyrate, 2-linoleoylglycerophosphocholine and palmitate (16:0). The results did not reach statistical significance following FDR correction (*q* values > 0.1). The results for horizontal pleiotropy using the MR-Egger regression intercept (*p* values > 0.05), for heterogeneity using Cochran’s Q statistic (*p* values > 0.05), and for outliers using MR-PRESSO (global test *p* values > 0.05) showed no significant differences (Table S6). The leave-one-out analysis results of cytokines and metabolites (Figure S2) suggested stable results.

### 3.3. Effect of GM on Cytokines and Metabolites

Based on the initial findings, we studied the relationship between the 18 gut microbial taxa and 1 cytokine or 13 metabolites (Table S7). The results indicated that *Eggerthella* genus was linked to an increased level of CTACK (OR = 1.15, 95% CI: 1.02–1.29, *p* = 0.021). The analysis revealed that Proteobacteria phylum was associated with an increased level of 10-undecenoate (OR = 1.03, 95% CI: 1.00–1.05, *p* = 0.037). *Coprobacter* genus showed a correlation with elevated 2-aminobutyrate level (OR = 1.02, 95% CI: 1.00–1.03, *p* = 0.012) and also with increased 3-(4-hydroxyphenyl) lactate level (OR = 1.02, 95% CI: 1.01–1.04, *p* = 0.005). *P. johnsonii* species was linked to an increased level of indolepropionate (OR = 1.02, 95% CI: 1.00–1.04, *p* = 0.045). *S. parasanguinis* species was linked to an increased level of 2-aminobutyrate (OR = 1.02, 95% CI: 1.00–1.03, *p* = 0.035). Conversely, *Paraprevotella* genus was linked to a decreased level of hypoxanthine (OR = 0.99, 95% CI: 0.97–1.00, *p* = 0.037) and a decreased level of phenylalanylphenylalanine (OR = 0.97, 95% CI: 0.95–0.99, *p* = 0.004). *RuminococcaceaeUCG005* genus demonstrated an association with a reduced level of 3-(4-hydroxyphenyl) lactate (OR = 0.97, 95% CI: 0.94–1.00, *p* = 0.046). *B. salyersiae* species was connected to a decreased p-acetamidophenylglucuronide level (OR = 0.82, 95% CI: 0.70–0.96, *p* = 0.015) and *R. callidus* species was related to lower hypoxanthine level (OR = 0.96, 95% CI: 0.93–0.98, *p* = 0.002). The results showed no significant differences in horizontal pleiotropy (MR-Egger regression intercept, *p* > 0.05), heterogeneity (Cochran’s Q, *p* > 0.05), and outliers (MR-PRESSO global test, *p* > 0.05) (Table S8).

### 3.4. Reverse Mendelian Randomization Analysis Results

In the reverse MR analysis (Table S9), HIV infection was set as the exposure, while gut microbial taxa, inflammatory cytokines and metabolites were set as outcomes. The results showed no significant reverse causal relationships between these variables (*p* values > 0.05). A significant association was found between HIV infection and 10-undecenoate (11:1n1) using IVW methods (*p* < 0.05) in the reverse MR analysis, leading to the exclusion of 10-undecenoate (11:1n1) from further analyses. Additionally, no reverse causal relationships were detected between CTACK, 12 metabolites, and 18 gut microbial taxa (*p* values > 0.05).

### 3.5. Mediation Analysis Results

Using cytokine (CTACK) and 12 metabolites as mediators, mediation relationships between GM and HIV infection were evaluated using the product of coefficients method (Table S10). The effect of *R. callidus* species on HIV infection was partly mediated by hypoxanthine, with a mediated effect β of 0.17 (95% CI: 0.01–0.34, *p* = 0.042). However, this association did not survive correction for multiple testing using the Benjamini–Hochberg procedure (*q* = 0.153).

### 3.6. External Association Based on 16S rRNA Gene Sequencing

**Figure 4:**
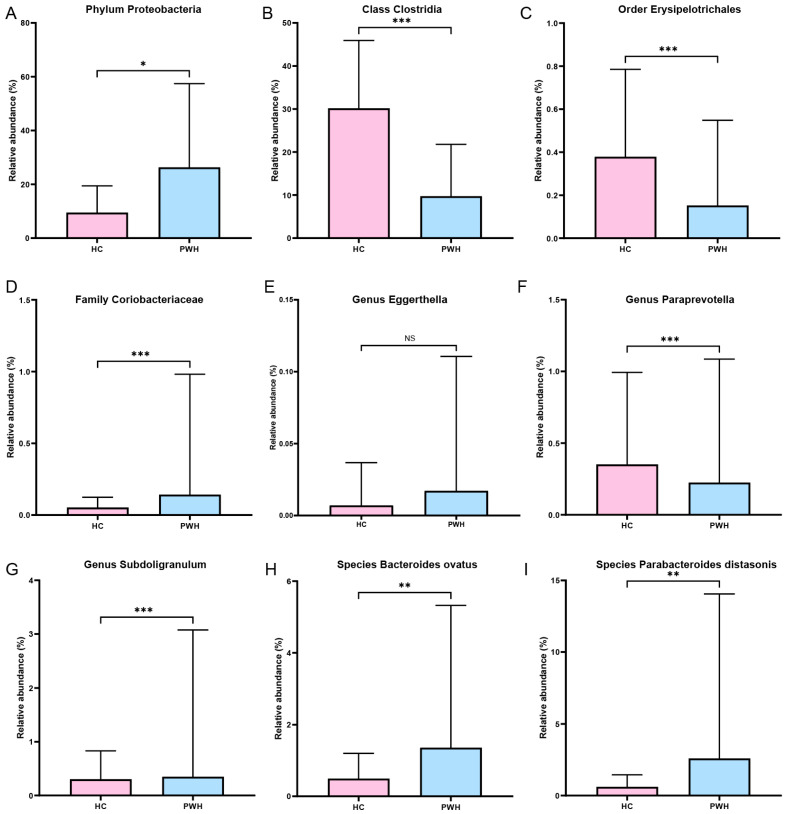
Comparison of the relative abundance of the nine target gut microbiota taxa between the HC and PWH groups based on 16S rRNA gene sequencing data. (**A**) Proteobacteria phylum; (**B**) Clostridia class; (**C**) Erysipelotrichales order; (**D**) *Coriobacteriaceae* family; (**E**) *Eggerthella* genus; (**F**) *Paraprevotella* genus; (**G**) *Subdoligranulum* genus; (**H**) *Bacteroides ovatus* species); (**I**) *Parabacteroides distasonis* species. * indicates *p* < 0.05, ** indicates *p* < 0.01, *** indicates *p* < 0.001; “NS” indicates not significant.

**Figure 5:**
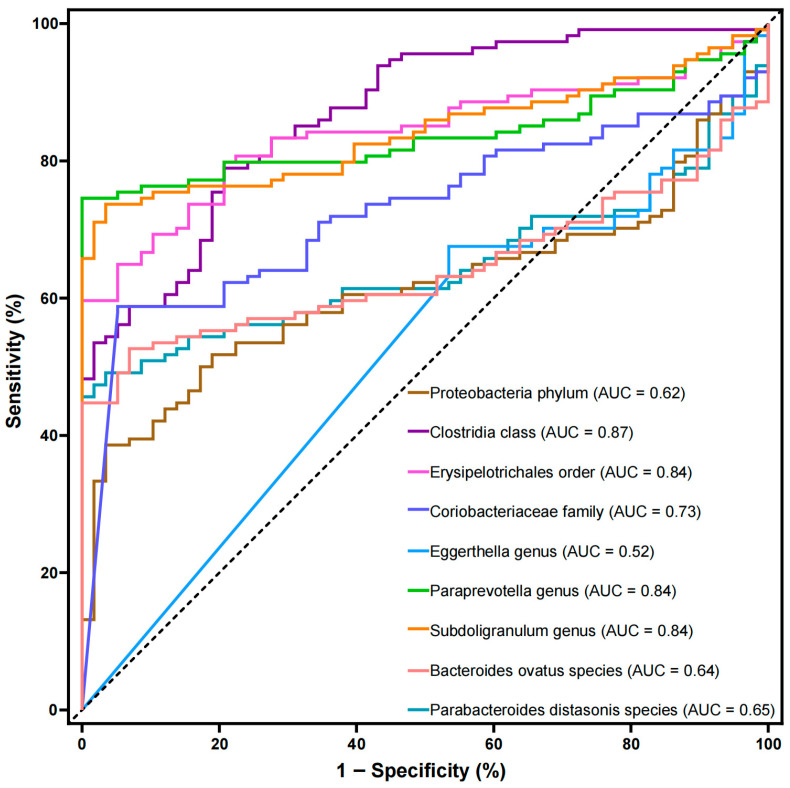
Prediction by nine microbial biomarkers between the HC and PWH groups. The results of the area under the receiver operating characteristic (ROC) curve for each taxon are plotted with different colors.

Among the 18 gut microbial taxa identified by the MR analysis results as potentially linked to HIV infection, 16 taxa were detected in the 16S rRNA gene sequencing data. After excluding seven taxa (Bacillales order, *Coprobacter* genus, and *B. salyersiae*, *P. johnsonii*, *P. capillosus*, *R. callidus* and *S. parasanguinis* species) with low relative abundance (Figure S3), the remaining nine taxa were selected for external assessment. The Mann–Whitney U test was used to assess the proportional differences between the HC and PWH groups. Compared to the HC group (Figure 4), the PWH groups showed significantly lower proportions of Clostridia class, Erysipelotrichales order, and *Paraprevotella* genus, and higher proportions of Proteobacteria phylum, *Coriobacteriaceae* family, *Subdoligranulum* genus, *B. ovatus*, and *P. distasonis* (*p* values < 0.05). However, no significant difference was found in the relative abundance of *Eggerthella* genus between the two groups (*p* = 0.70). Eight significantly different taxa, as biomarkers, effectively distinguished the HC and PWH groups (Table 1 and Figure 5). The AUC values for differentiating HC and PWH groups were as follows: Proteobacteria phylum, 0.62 (95% CI: 0.53–0.70, *p* = 0.013); Clostridia class, 0.87 (95% CI: 0.82–0.92, *p* < 0.001); Erysipelotrichales order, 0.84 (95% CI: 0.78–0.90, *p* < 0.001); *Coriobacteriaceae* family, 0.73 (95% CI: 0.65–0.80, *p* < 0.001); *Paraprevotella* genus, 0.84 (95% CI: 0.78–0.90, *p* < 0.001); *Subdoligranulum* genus, 0.84 (95% CI: 0.78–0.90, *p* < 0.001); *B. ovatus* species, 0.64 (95% CI: 0.56–0.72, *p* = 0.002); and *P. distasonis* species, 0.65 (95% CI: 0.57–0.73, *p* = 0.002).

## 4. Discussion

The intricate composition of the GM plays a key role in both host health and diseases. HIV infection risk is consistently associated with gut microbiota dysbiosis in multiple observational studies [40,41,42]. However, the available evidence is limited by confounding factors and the potential for reverse causation. Furthermore, the extrapolation of results is complicated by findings from animal models, which may not mirror human microbial ecology. As reported by multiple studies, a prominent HIV-associated microbiome alteration is characterized by the enrichment of *Prevotella* alongside the depletion of *Bacteroides* [43,44]. However, Noguera-Julian et al. demonstrated that the elevated *Prevotella*/*Bacteroides* ratio is associated with specific behavioral patterns among men who have sex with men (MSM), independent of HIV infection status [5]. In contrast to earlier observational studies, our research integrates a TSMR framework with publicly accessible GWAS datasets to comprehensively evaluate the potential causal relationship between the GM and HIV infection. This approach effectively controls for confounding biases and reverse causality, thereby enhancing the accuracy of these results. This study represents the first application of MR analysis to investigate the associations between GM and HIV infection at the species level, leveraging publicly available GWAS data. In order to ensure the reliability of the findings, 16S rRNA gene sequencing was utilized as a supplementary method. This multidimensional approach supports the potential involvement of GM in HIV infection, providing a theoretical foundation for subsequent mechanistic studies and precision interventions.

In our study, we observed suggestive associations between 18 gut microbial taxa and HIV infection, with 9 taxa showing negative associations and 9 showing positive associations. These findings suggest that gut microbiota composition may differentially modulate host susceptibility to HIV, with distinct taxonomic groups potentially exerting opposing effects. Following the conversion of OR to β coefficient [β = ln (OR)], the estimated effects of GM on HIV infection exhibited absolute values of β coefficients (|β|) ranging from 0.254 to 1.318. The majority of these 18 taxa belonged to Bacillota phylum. These findings align closely with previous reports at the genus level or higher taxa, further supporting the substantial role of GM dysbiosis in HIV infection [21,22]. For example, *Subdoligranulum* genus was reported to be enriched in individuals with HIV, aligning with its positive association in our analysis [45]. Interestingly, whereas *R. Callidus* was negatively associated with HIV infection, the butyrate-producing bacteria *RuminococcaceaeUCG005* showed a positive association. This apparent discrepancy underscores the complex interactions within the gut microbiota, suggesting that its influence on HIV infection cannot be attributed to a single microbial species. These findings highlight the need for further investigation into the diversity and composition of gut bacteria. At the species level, we observed three species negatively associated with HIV infection and four positively associated. *P. distasonis* is a beneficial symbiotic bacterium that enhances intestinal barrier function and mucosal immune homeostasis by producing metabolites such as short-chain fatty acids, which affect the inflammatory microenvironment [46,47]. Our study enriches the current limited understanding of the role of gut microbiota dysbiosis at the species level in relation to HIV infection. Among the remaining species, those associated with HIV infection risk have not been reported in previous case–control studies. Further research is warranted to elucidate the biological roles of these taxa.

Among the 18 taxa identified by the MR analysis, 7 taxa were able to effectively distinguish between HC and PWH groups in our 16S rRNA gene sequencing data, suggesting their potential value as microbial biomarkers. However, one taxon, *P. distasonis*, exhibited directional inconsistency between the MR and 16S rRNA gene sequencing results. Specifically, the MR analysis result indicated that the genetically predicted abundance of *P. distasonis* was negatively associated with HIV infection, consistent with the previously reported beneficial function of this species [46]. In contrast, the relative abundance of *P. distasonis* was higher in the PWH group than in the HC group. MR leverages host genetic variants to infer associations between genetically predicted microbial abundance and HIV infection risk, whereas 16S rRNA gene sequencing captures the gut microbiota composition after HIV infection. In this post-infection state, CD4^+^ T cell depletion in the gut, disruption of the mucosal barrier, microbial translocation, and chronic inflammation collectively drive widespread dysbiosis, which may alter the abundance of otherwise potentially protective bacteria through shifts in ecological niches. Furthermore, due to the compositional nature of microbiota data, an increase in relative abundance does not necessarily reflect a true increase in absolute load but may instead result from the depletion of other taxa [48]. Future studies integrating longitudinal designs, shotgun metagenomic profiling, absolute quantification, and functional assays are needed to clarify the specific role of *P. distasonis* strains in HIV infection and disease progression, thereby bridging the gap between MR inferences and the observational ecological changes.

The MR analyses revealed the effects of HIV infection on one cytokine and 13 xenobiotic metabolites. The absolute values of β coefficients (|β|) were 0.381 for the cytokine and ranged from 0.059 to 4.198 for the xenobiotic metabolites. A previous study reported that for each average unit increase in CTACK, there is an increase in HIV hazard of 3.94% (HR: 1.039, 95% CI: 1.016–1.063). CTACK directs T-cells to the skin, indicating immune surveillance at mucosal and skin surfaces to prevent initial HIV infection [49]. The MR results via the IVW method showed 2-linoleoylglycerophosphocholine and palmitate (16:0) as positively associated with HIV infection risk, with ORs exceeding 20. Conversely, hypoxanthine, citrulline and phenylalanylphenylalanine were negatively associated with HIV infection risk, with ORs below 0.5. Hypoxanthine exhibits antiviral potential. After identifying human retroviruses like HIV-1, research efforts were carried out to identify drugs capable of treating or preventing lethal diseases caused by viruses, and it was shown that several nucleoside analogs exhibit in vitro anti-HIV-1 activity in accordance with the development of clinical trials in 1987 [50]. It was also found that a variety of purine derivatives had antiviral potential, such as hypoxanthine (6-hydroxypurine). In addition, this compound is an intermediate product in the synthesis of other substituted purines, such as 6-mercaptopurine, considered an antiviral agent of the purine series and derived from azathioprine [51]. However, evidence on its direct protective effects against HIV infection remains limited, necessitating further research into the role of hypoxanthine in HIV infection.

In our mediation analysis, we observed instances where the direct and indirect effects exhibited opposite directions. This phenomenon, termed “inconsistent mediation” in the statistical and epidemiological literature, is also methodologically referred to as a “suppression effect” [52,53]. It indicates that an exposure may influence an outcome through opposing pathways, thereby supporting the methodological validity of the mediation approach employed in our study. However, after correction for multiple testing using Benjamini–Hochberg, no statistically significant mediation effects (cytokines or metabolites) were identified in the pathway from gut microbiota to HIV infection. Regarding mediation, a prior study suggests metabolites and cytokines may mediate the relationship between GM and HIV infection [22]. However, our results indicated no causal link between 18 HIV-related taxa and corresponding blood metabolites or cytokines, which may stem from partial differences in the datasets utilized in our study compared to the prior one.

This study has several strengths. We used a TSMR design to control for confounding factors and minimize reverse causality concerns. We integrated the Lifelines cohort data with the widely used MiBioGen database, extending our results from genus-level and higher taxonomic ranks to the species level. This provides valuable genetic variation information for assessing the relationship between gut microbiota and HIV infection risk. Additionally, we conducted external assessment of the key gut microbiota using independent 16S rRNA gene sequencing data. This multi-omics strategy substantially enhances the robustness and credibility of our results. Nevertheless, there are several limitations in this study. Firstly, the sample size of GM GWAS remains modest and the number of loci examined was relatively limited. We used a lenient genome-wide significance threshold (*p* < 1 × 10^−5^) for screening HIV infection and gut microbiota-related SNPs. Despite implementing F-statistic calculations for each SNPs’ instrument validity, the possibility of false negative errors due to insufficient statistical power cannot be excluded. Both the GWAS data of HIV infection, which were primarily derived from European-ancestry populations, and the 16S rRNA gene sequencing cohort, recruited from a single center in our prior study, limit the generalizability of our findings to broader and more diverse populations. Detailed information on participants’ sexual behavior was not collected due to ethical and privacy considerations. The effect of antiretroviral therapy on gut microbiota in people with HIV was also not assessed. Furthermore, fecal samples only reflect the microbial composition of the colon and do not accurately represent the microbial composition of intestinal regions, which is a limitation of current data that future research should address. Future studies should integrate multi-omics data and include more diverse, multicenter cohorts to enable a more comprehensive understanding of the causal relationship between the gut microbiota and HIV infection.

## 5. Conclusions

This study comprehensively investigates the potential role of the GM in HIV infection, employing multiple methods that integrate Mendelian randomization analysis with 16S rRNA gene sequencing. The consistent results across methods underscore the robustness of our findings. Our results highlight eight specific gut taxa, with four taxa (Clostridia class, Erysipelotrichales order, *Paraprevotella* genus, and *Parabacteroides distasonis* species) showing negative associations and four others (Proteobacteria phylum, *Coriobacteriaceae* family, *Subdoligranulum* genus, and *Bacteroides ovatus* species) showing positive associations with HIV infection risk. Furthermore, the potential of hypoxanthine as a mediator in the relationship between *R*. *callidus* and HIV infection was found. These insights provide a foundation for the development of more effective HIV prevention strategies through GM regulation and emphasize the necessity for further research to elucidate the mechanisms underlying these associations.

## Figures and Tables

**Table 1:** The AUC values for differentiating HC and PWH groups.

Gut Microbiota	AUC	95% CI	*p*
Proteobacteria phylum	0.62	0.53–0.70	0.013
Clostridia class	0.87	0.82–0.92	<0.001
Erysipelotrichales order	0.84	0.78–0.90	<0.001
*Coriobacteriaceae* family	0.73	0.65–0.80	<0.001
*Eggerthella* genus	0.52	0.43–0.61	0.6927
*Paraprevotella* genus	0.84	0.78–0.90	<0.001
*Subdoligranulum* genus	0.84	0.78–0.90	<0.001
*Bacteroides ovatus* species	0.64	0.56–0.72	0.002
*Parabacteroides distasonis* species	0.65	0.57–0.73	0.002

## Data Availability

The original contributions presented in this study are included in the article/Supplementary Material. Further inquiries can be directed to the corresponding authors.

## Supplementary Materials

The following supporting information can be downloaded at https://www.mdpi.com/article/10.3390/microorganisms14030667/s1: Figure S1: Leave-one-out analysis results of the significant causal effects of 18 gut microbiota on HIV infection. The X-axis indicates the estimated β value. In each panel, the red line stands for the overall estimates, and each black line indicates the overall estimate after excluding the left SNP. (A) Proteobacteria phylum; (B) Clostridia class; (C) Bacillales order; (D) Erysipelotrichales order; (E) *Coriobacteriaceae* family; (F) *Coprobacter* genus; (G) *Coprococcus2* genus; (H) *Eggerthella* genus; (I) *Paraprevotella* genus; (J) *RuminococcaceaeUCG005* genus; (K) *Subdoligranulum* genus; (L) *Bacteroides ovatus* species; (M) *Bacteroides salyersiae* species; (N) *Parabacteroides distasonis* species; (O) *Parabacteroides johnsonii* species; (P) *Pseudoflavonifractor capillosus* species; (Q) *Ruminococcus callidus* species; (R) *Streptococcus parasanguinis* species; Figure S2: Leave-one-out analysis results of the significant causal effects of 2 cytokines and 13 metabolites on HIV infection. The X-axis indicates the estimated β value. In each panel, the red line represents the overall estimate, and each black line shows the overall estimate after excluding the left SNP. Cytokines include (A) CTACK and (B) IL-17. Xenobiotics metabolites include (C) 10-undecenoate (11:1n1); (D) 1-myristoylglycerophosphocholine; (E) 2-aminobutyrate; (F) 2-linoleoylglycerophosphocholine; (G) 3-(4-hydroxyphenyl) lactate; (H) citrulline; (I) hypoxanthine; (J) indolepropionate; (K) lactate; (L) N-acetylglycine; (M) p-acetamidophenylglucuronide; (N) palmitate (16:0) and (O) phenylalanylphenylalanine; Figure S3: The low relative abundance of seven gut microbiota taxa based on 16S rRNA gene sequencing data. (A) Bacillales order; (B) *Coprobacter* genus; (C) *Bacteroides salyersiae* species; (D) *Parabacteroides johnsonii* species; (E) *Pseudoflavonifractor capillosus* species; (F) *Ruminococcus callidus* species and (G) *Streptococcus parasanguinis* species; Table S1: Data sources in this study; Table S2: Instruments variables for gut microbiota taxa selected at the genome-wide significance threshold (*p* < 5 × 10^−8^); Table S3: The causal relationships between gut microbiota and HIV infection with the UVMR analysis method; Table S4: The sensitivity analysis results between gut microbiota and HIV infection with MR Egger regression, Cochran’s IVW Q test, and MR-PRESSO analysis. MR, Mendelian randomization; SD, standard deviation; RSSobs, observed residual sum of squares; SNP, single nucleotide polymorphism; “-” indicate that there are not enough SNPs to analyze; Table S5: The causal relationships between cytokines or metabolites and HIV infection with the UVMR analysis method; Table S6: The sensitivity analysis results between cytokines or metabolites and HIV infection with MR Egger regression, Cochran’s IVW Q test, and MR-PRESSO analysis; Table S7: The causal relationships between gut microbiota taxa and cytokines or metabolites with the UVMR analysis method; Table S8: The sensitivity analysis results between gut microbiota taxa and cytokines or metabolites with MR Egger regression, Cochran’s IVW Q test, and MR-PRESSO analysis; Table S9: Results of reverse MR for significant causal relationships between HIV infection and gut microbiota, cytokines and metabolites; Table S10: Mediation analysis results.

## Abbreviations

The following abbreviations are used in this manuscript:

GM	Gut microbiota
MR	Mendelian randomization
HC	Healthy control
PWH	People with HIV
AUC	Area under the curve
HIV	Human immunodeficiency virus
AIDS	Acquired immunodeficiency syndrome
IVs	Instrumental variables
TSMR	Two-sample Mendelian randomization
GWAS	Genome-wide association study
SNPs	Single-nucleotide polymorphisms
DMP	Dutch Microbiome Project
LD	Linkage disequilibrium
MR-PRESSO	Mendelian Randomization Pleiotropy Residual Sum and Outlier
IVW	Inverse-variance weighted
WME	Weighted median estimator
SM	Simple mode
WM	Weighted mode
ORs	Odds ratios
CI	Confidence intervals
UVMR	Univariable mendelian randomization
