# Supplementary figures and images for: Association Between Gut Microbiota and HIV Infection Risk: Insights from Mendelian Randomization and 16S rRNA Amplicon Sequencing
Source: Microorganisms. 2026 Mar 15;14(3):667. doi: 10.3390/microorganisms14030667 (PMC13029254; doi:10.3390/microorganisms14030667)

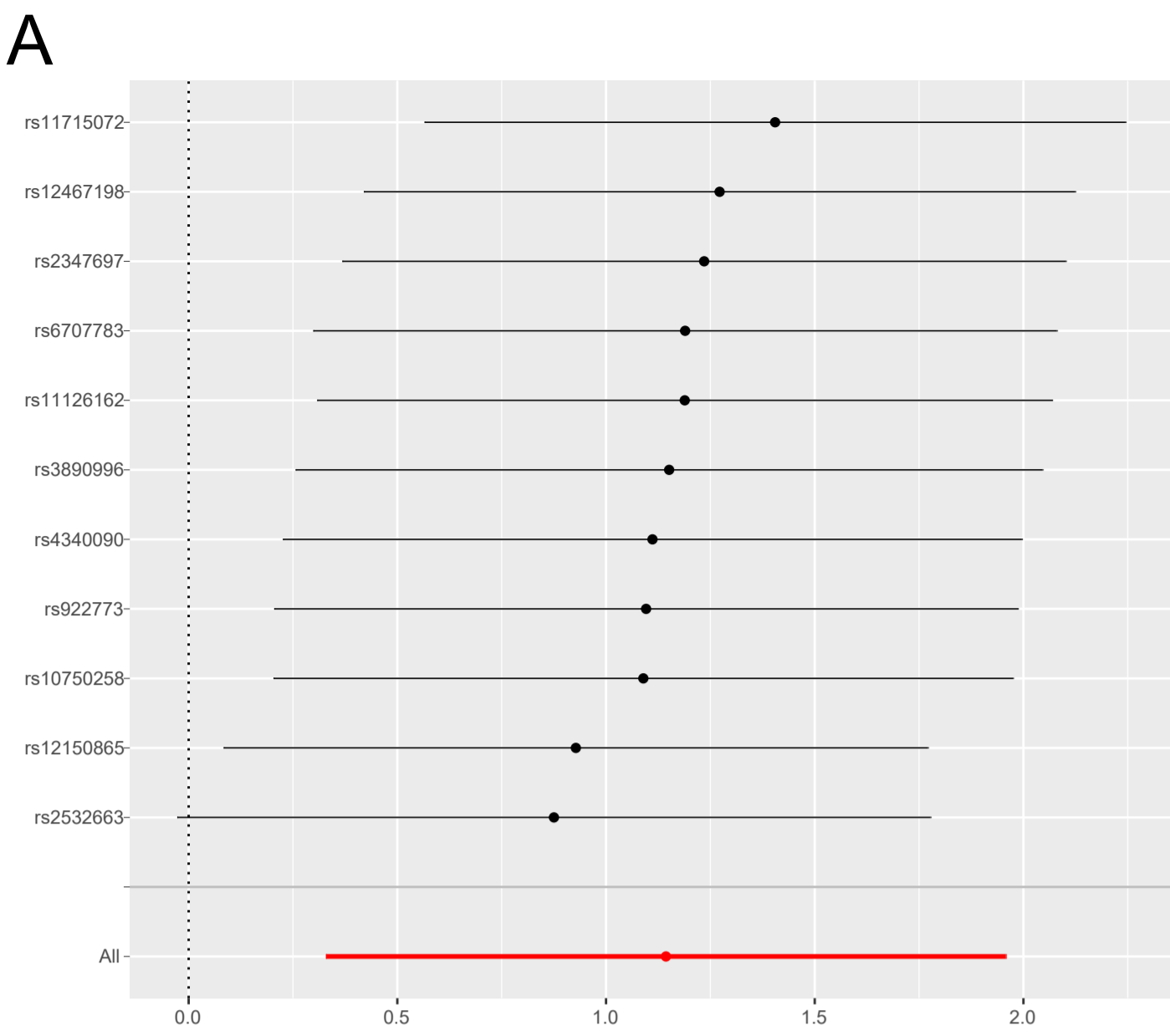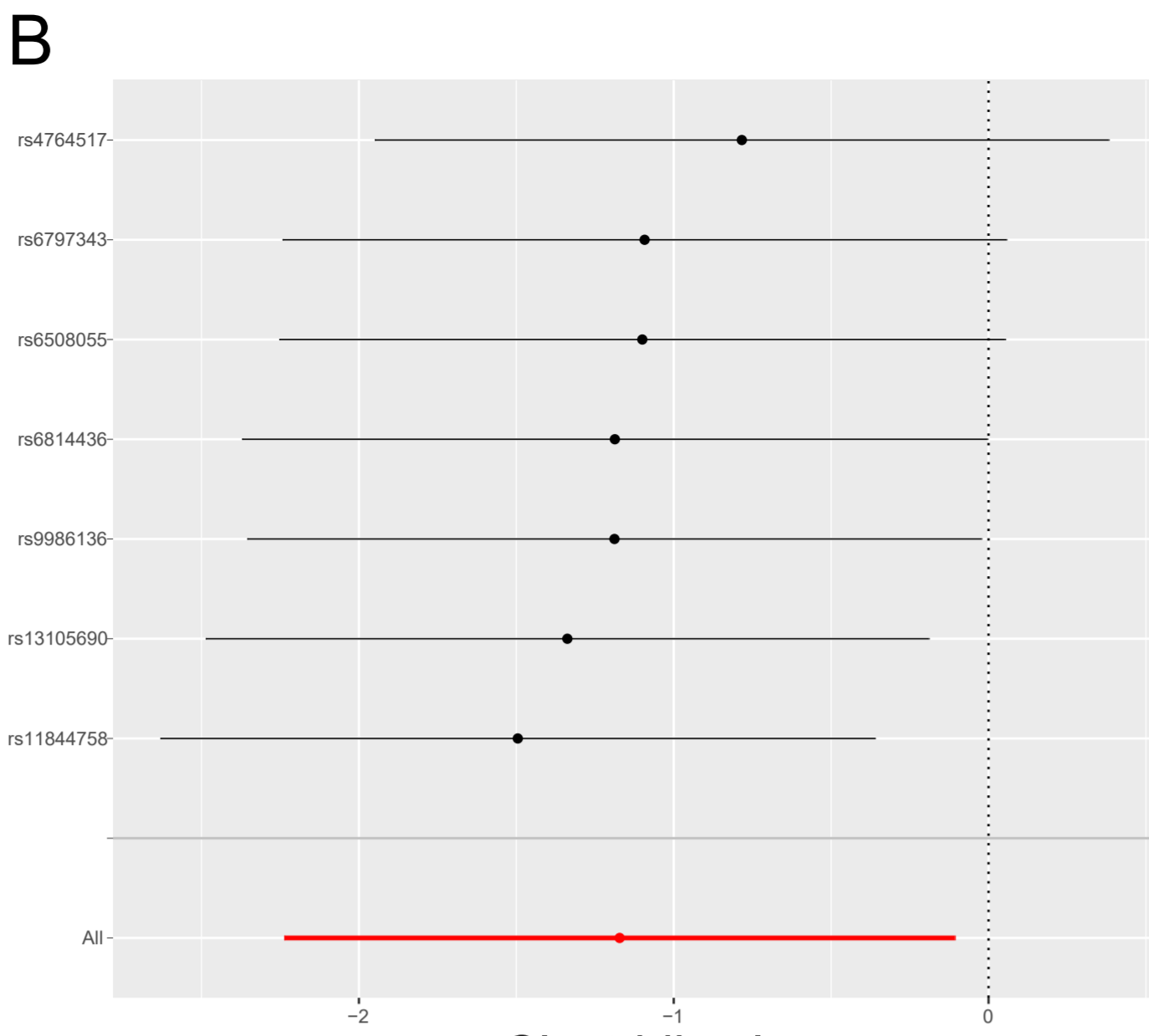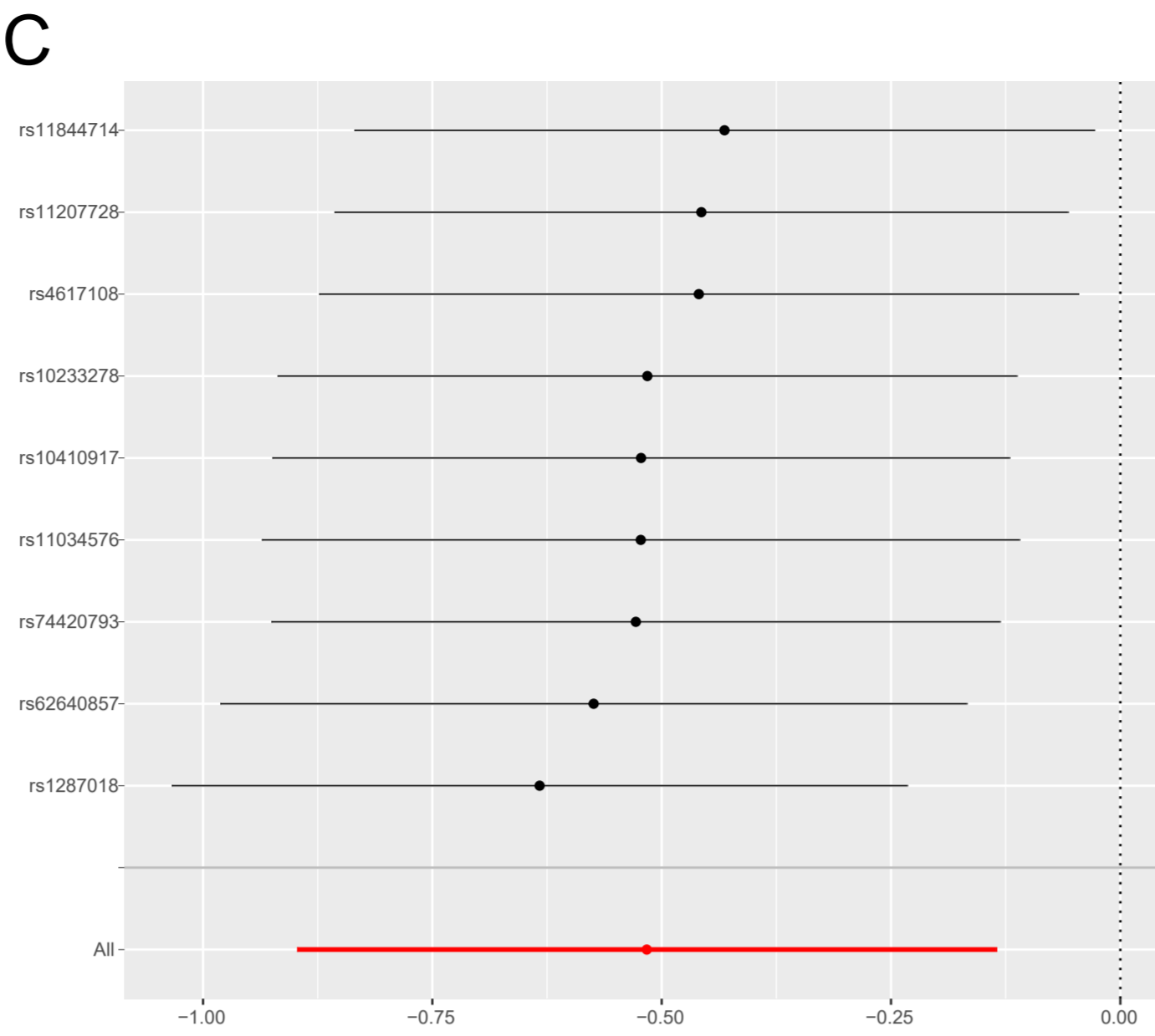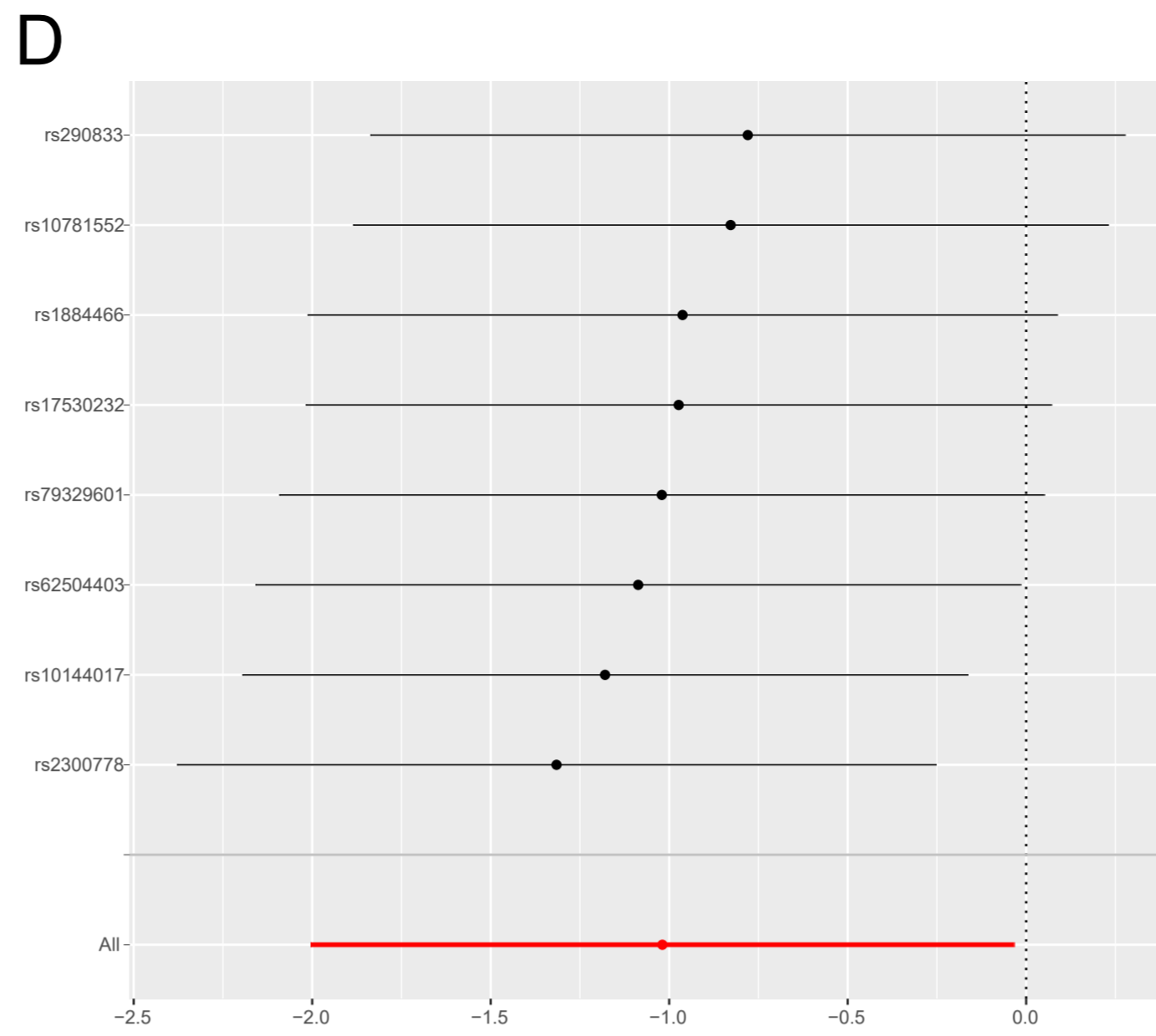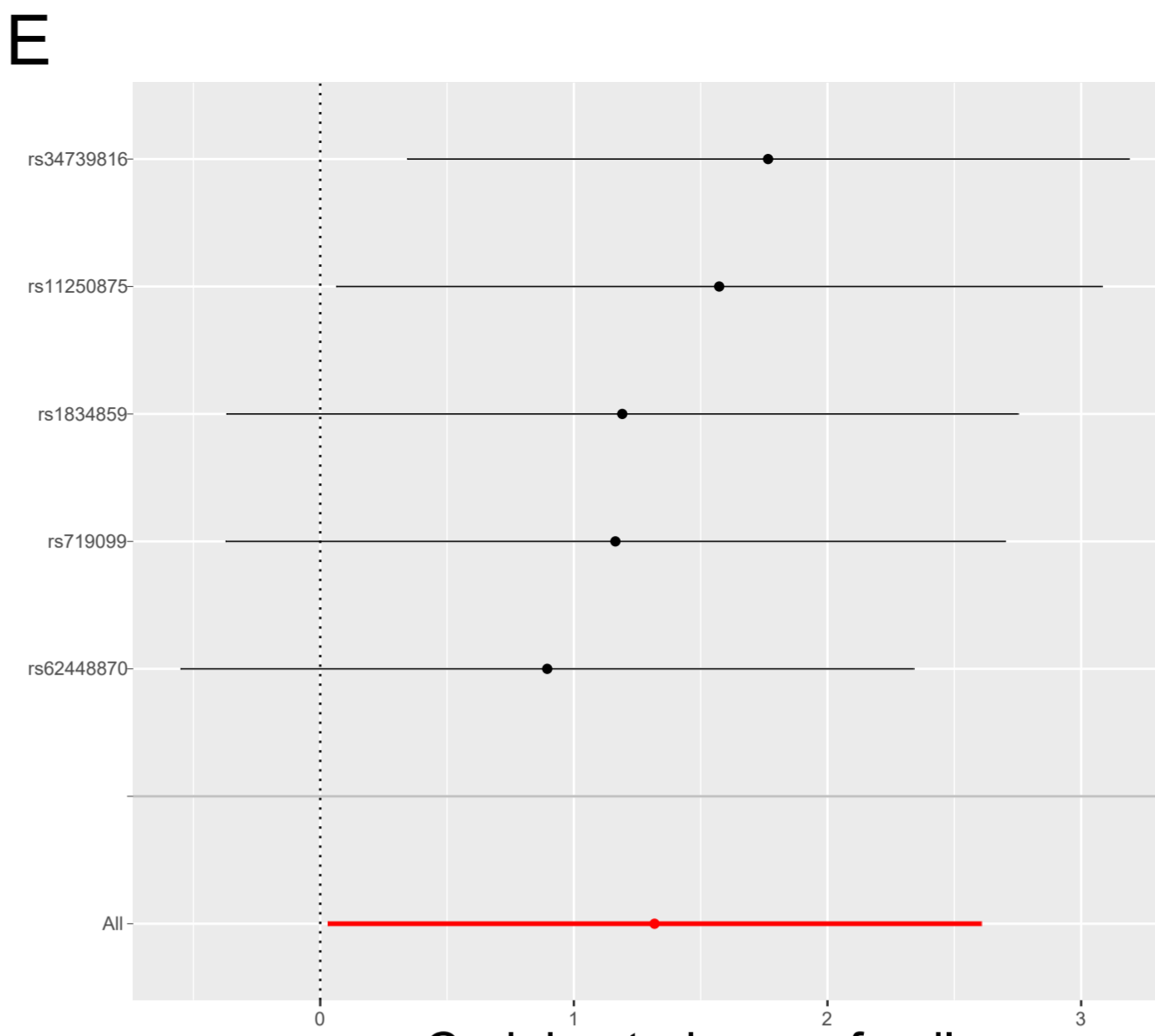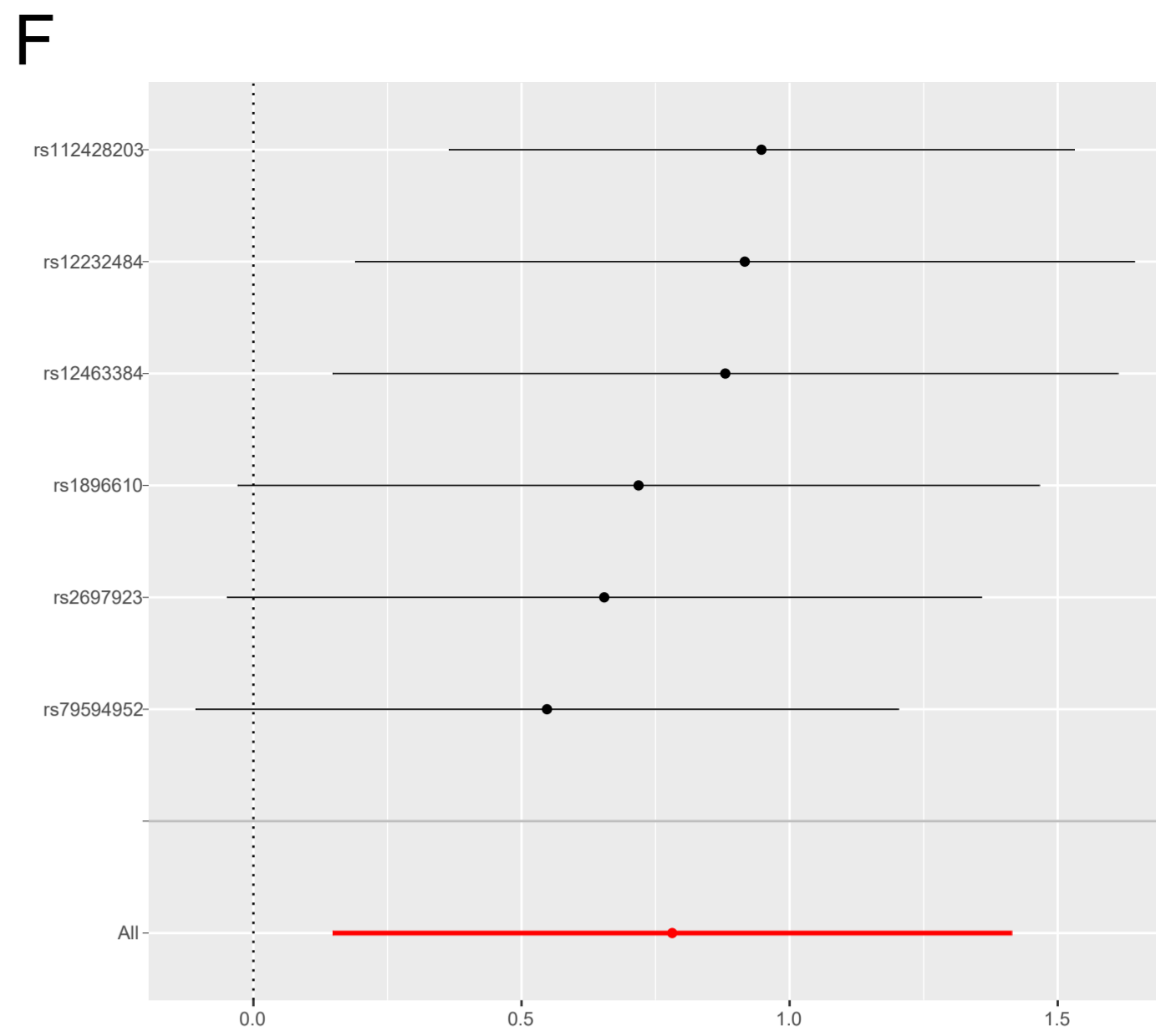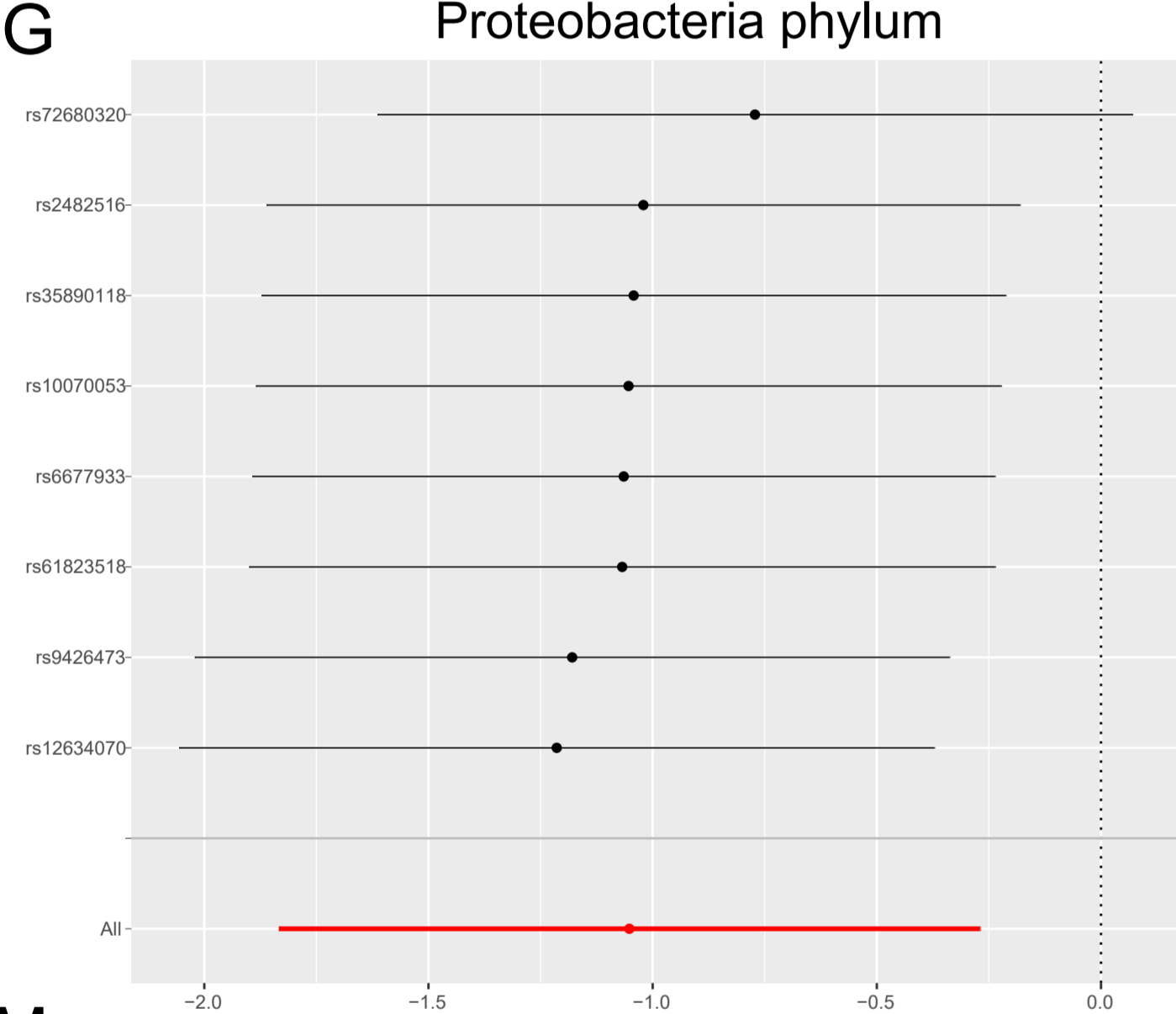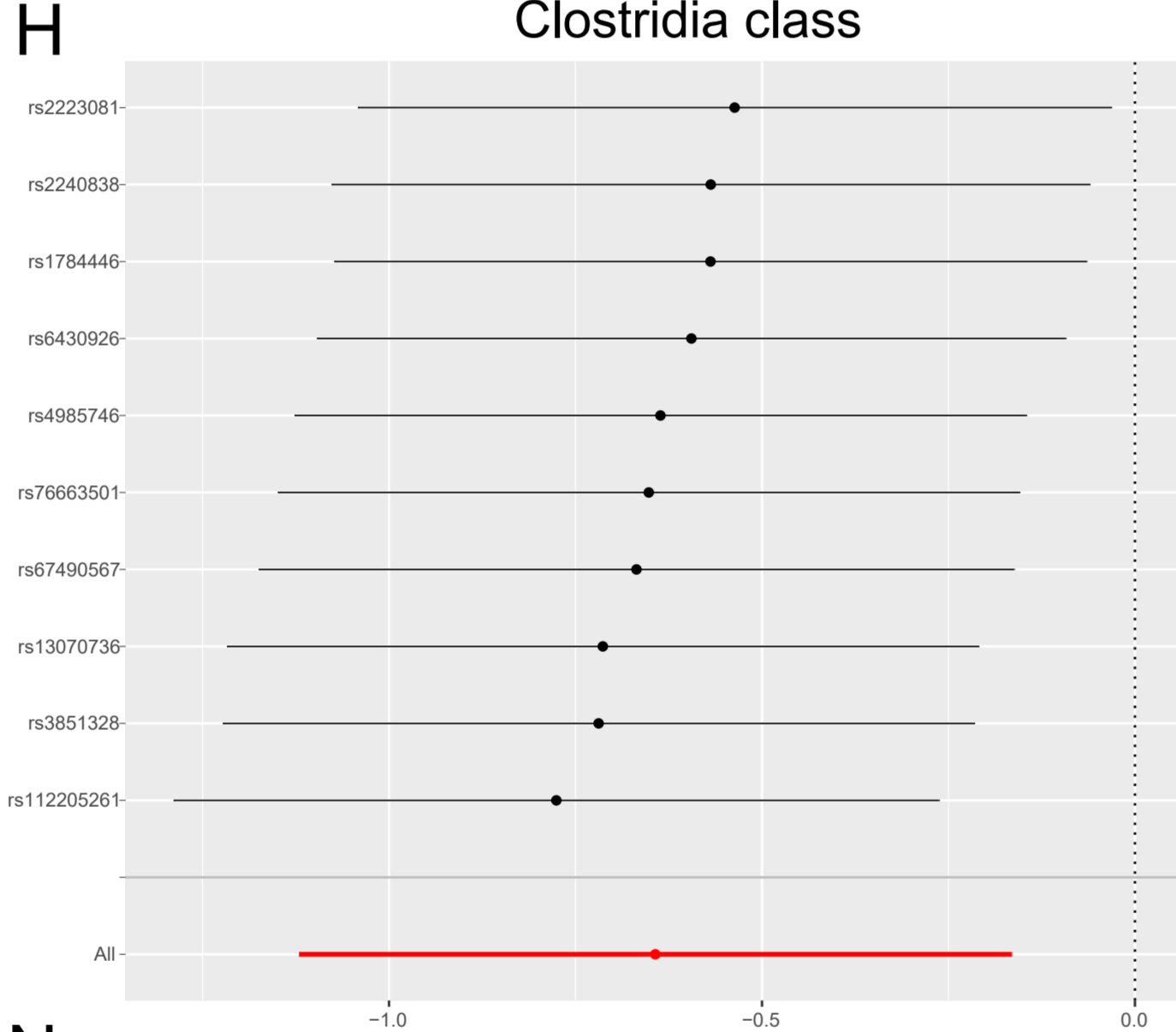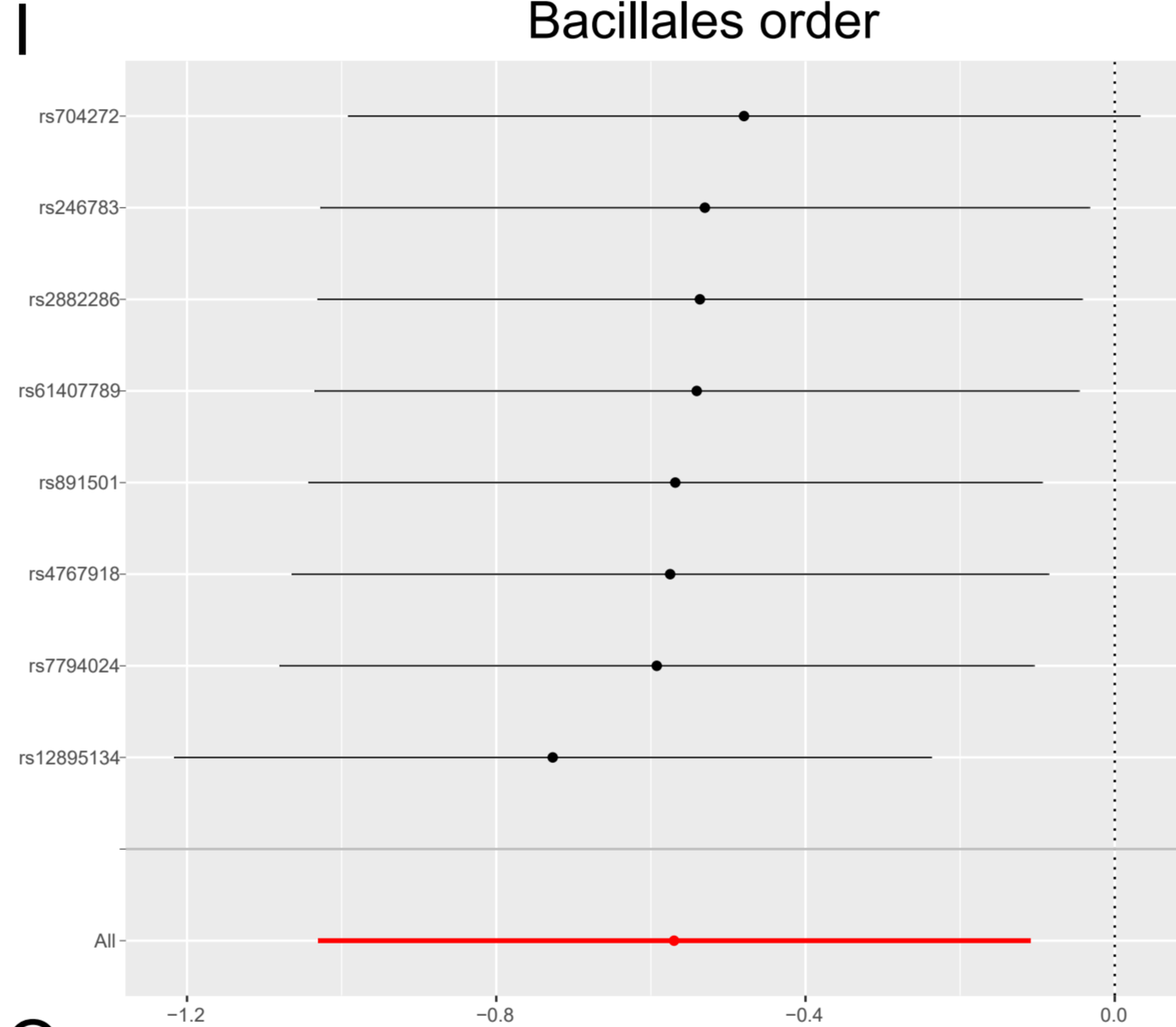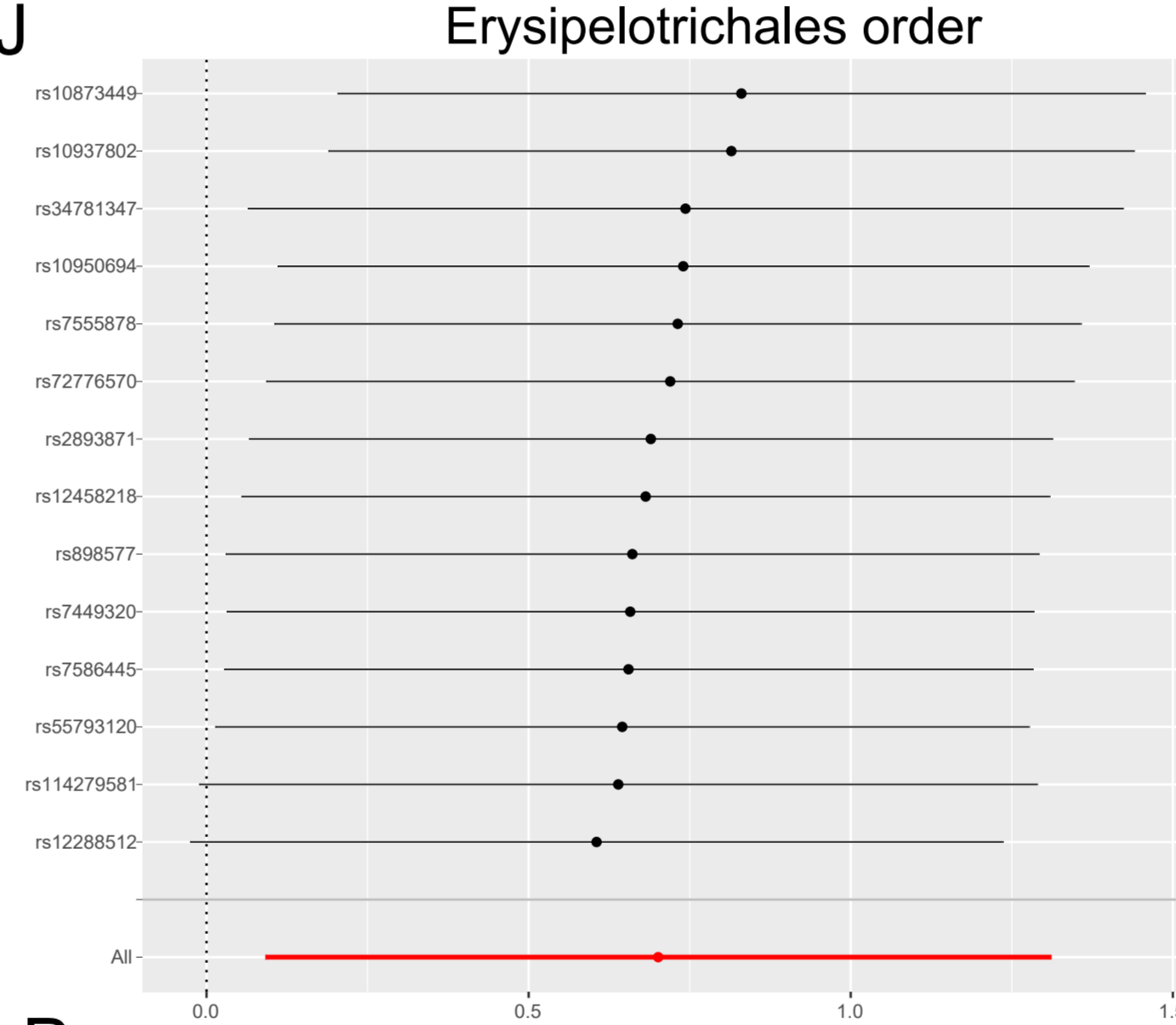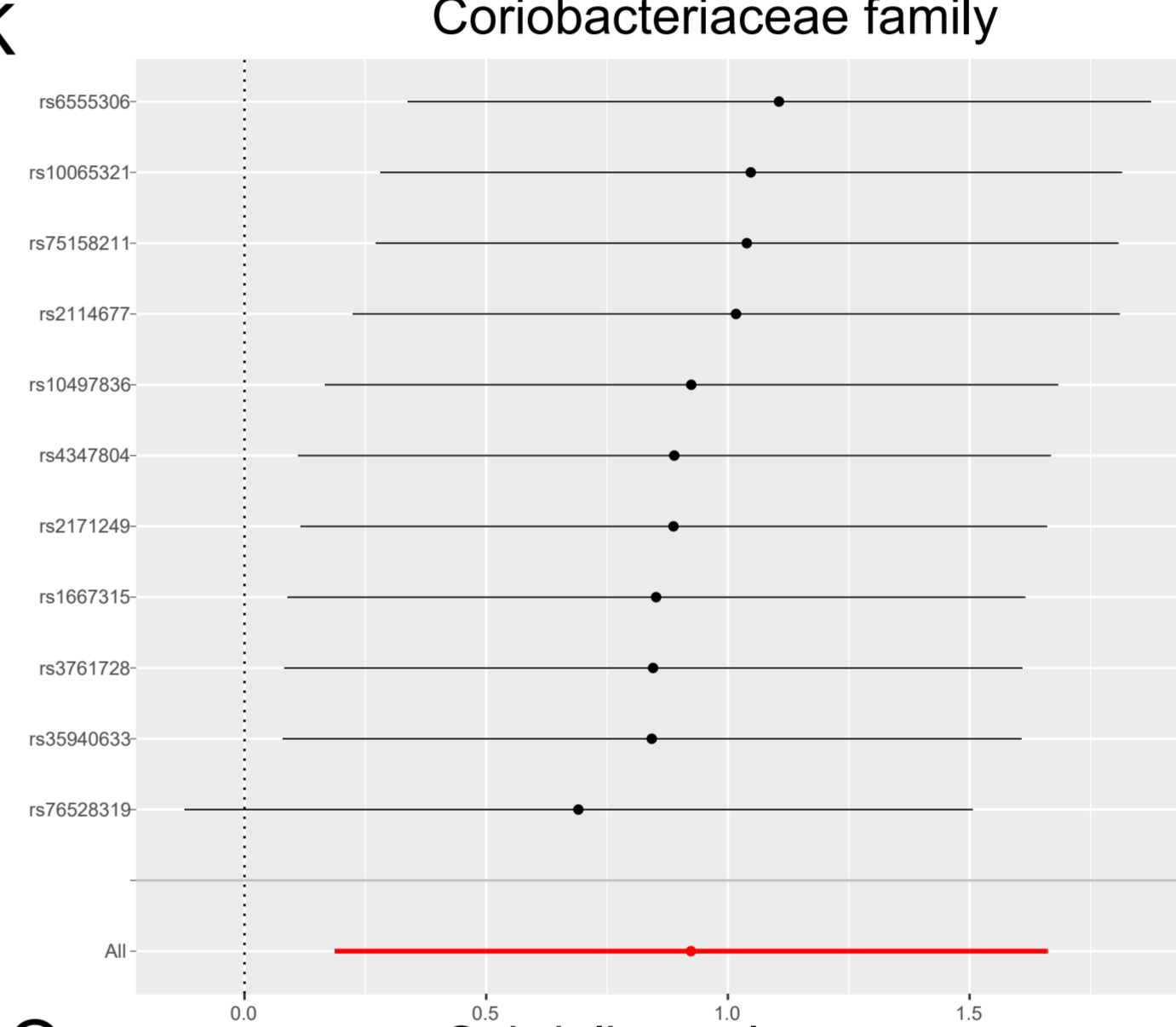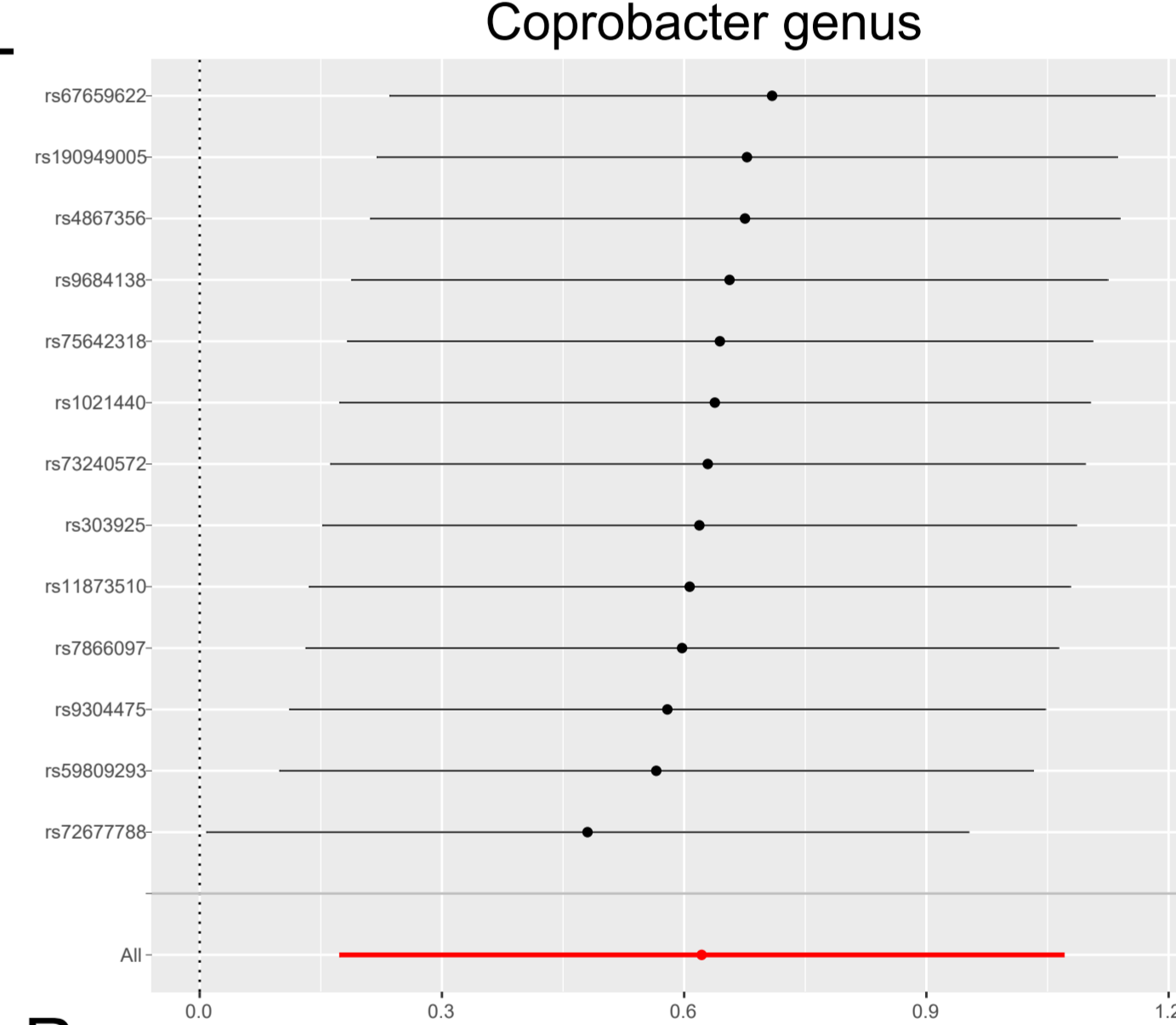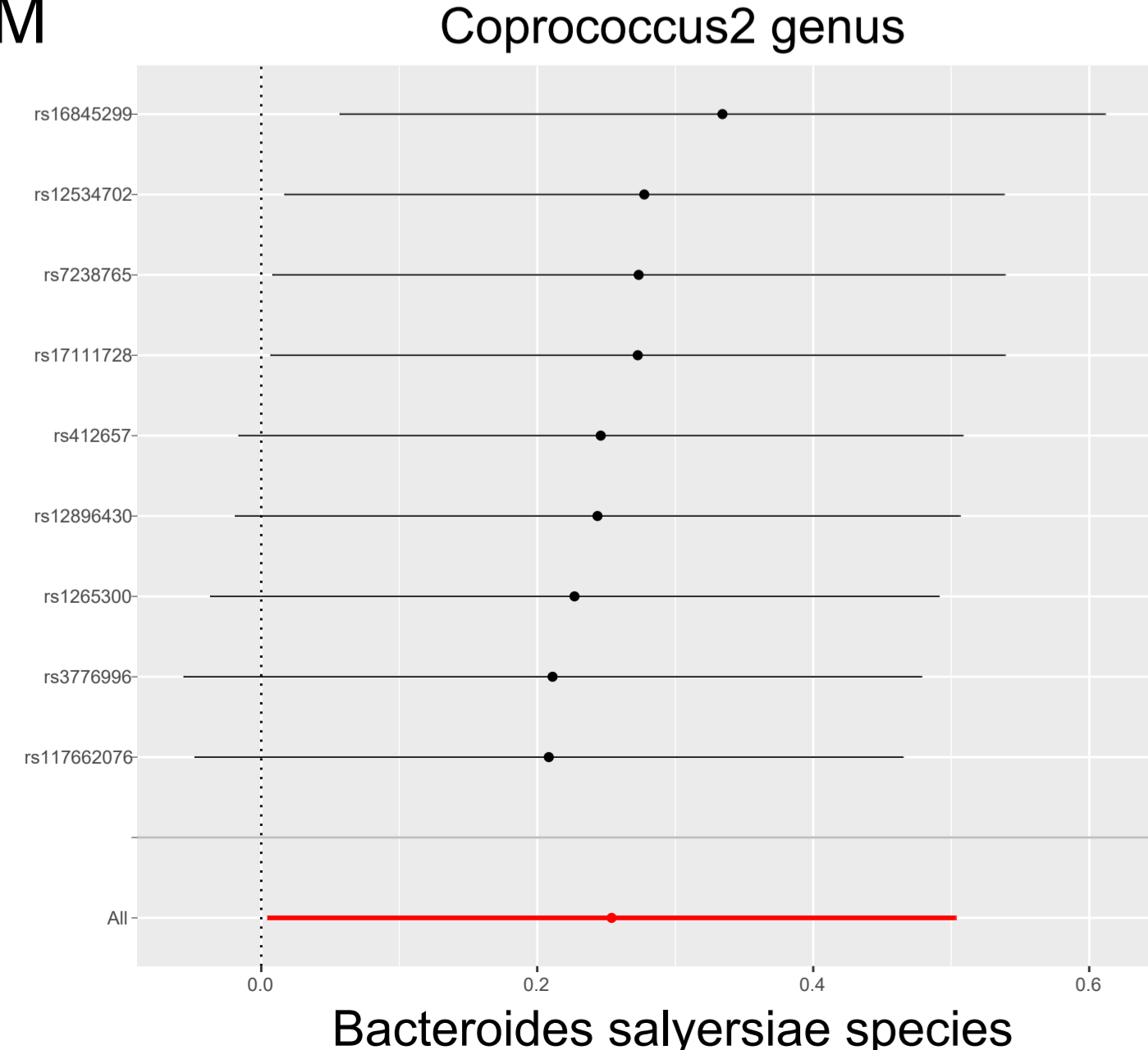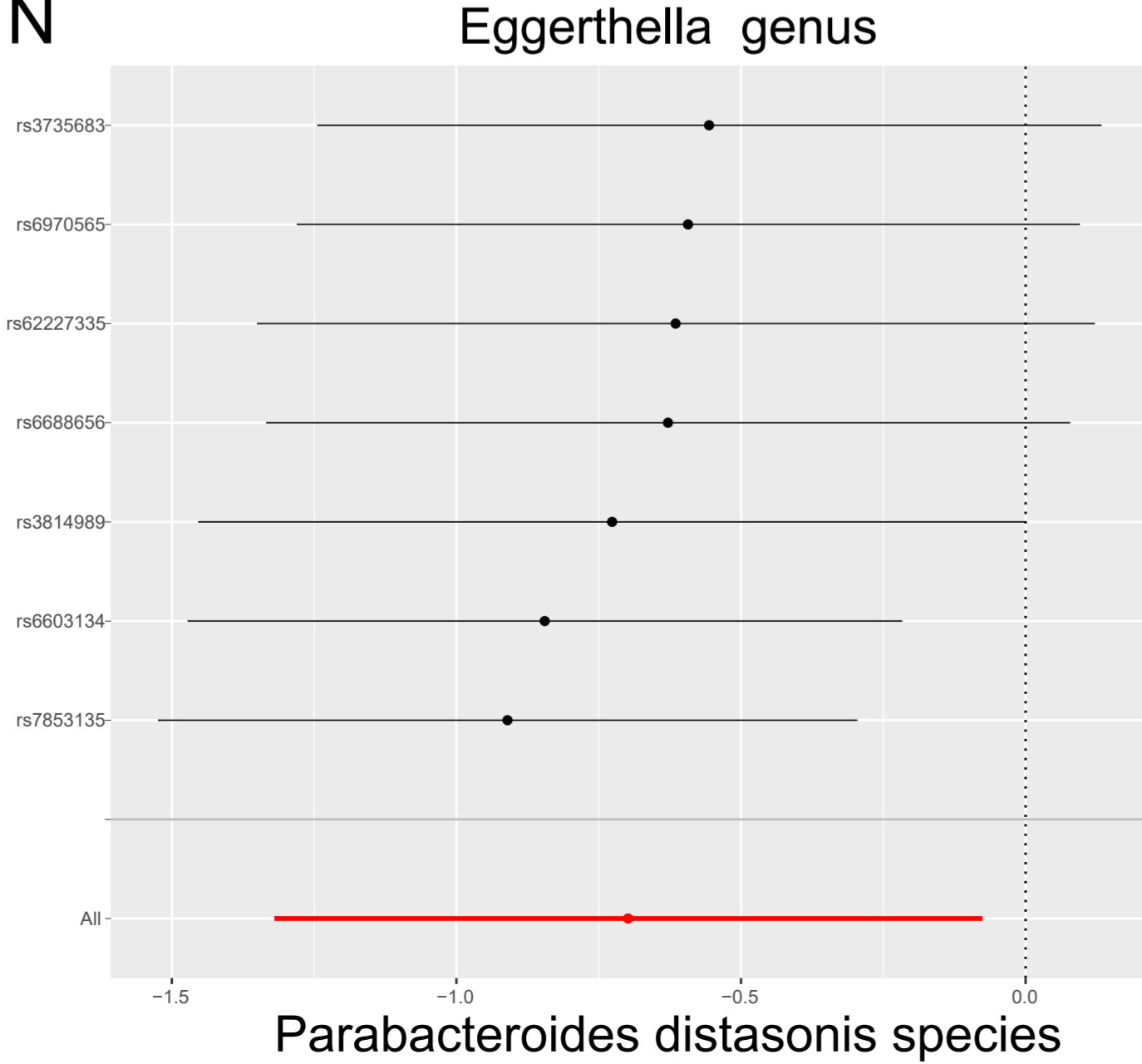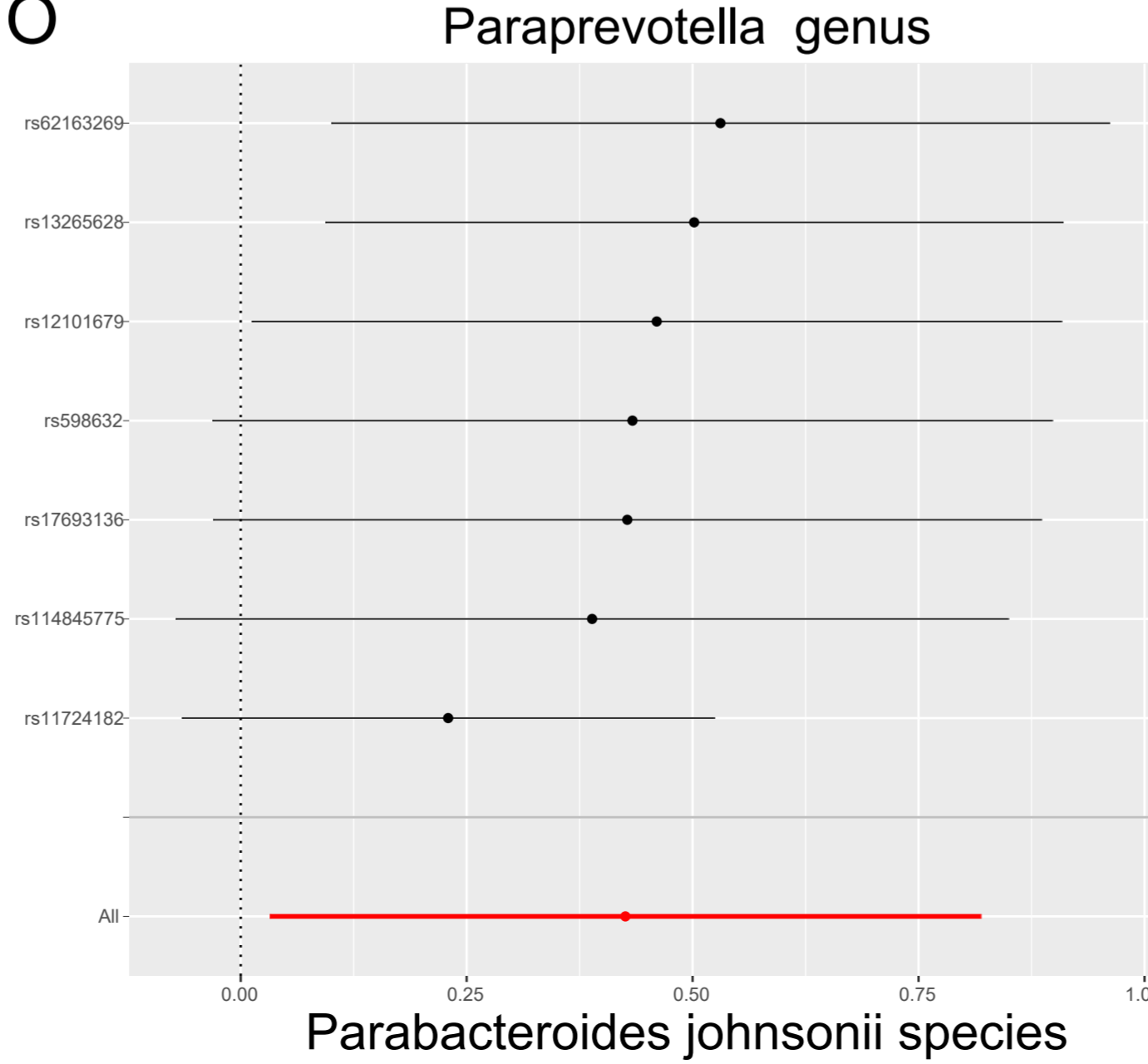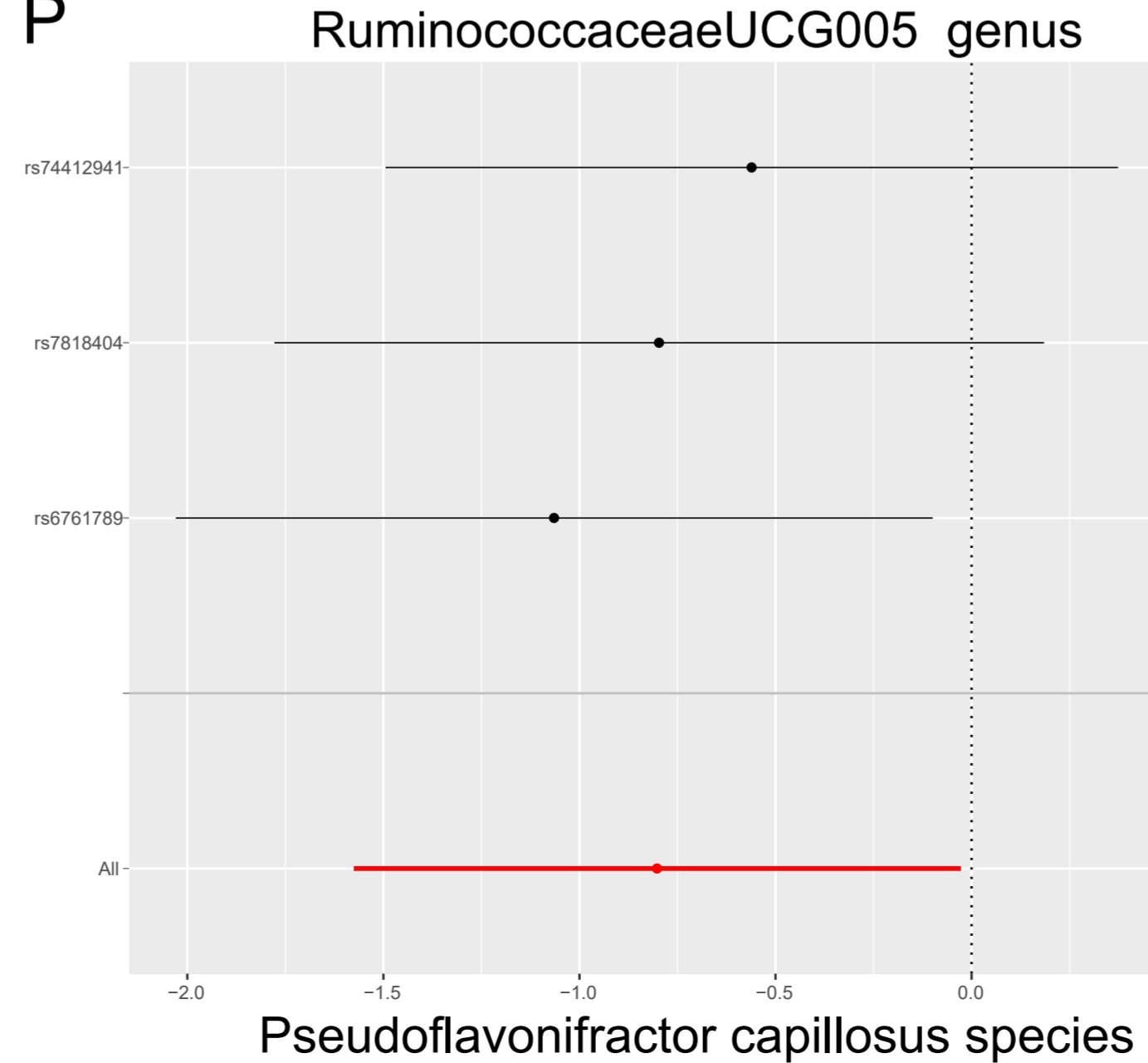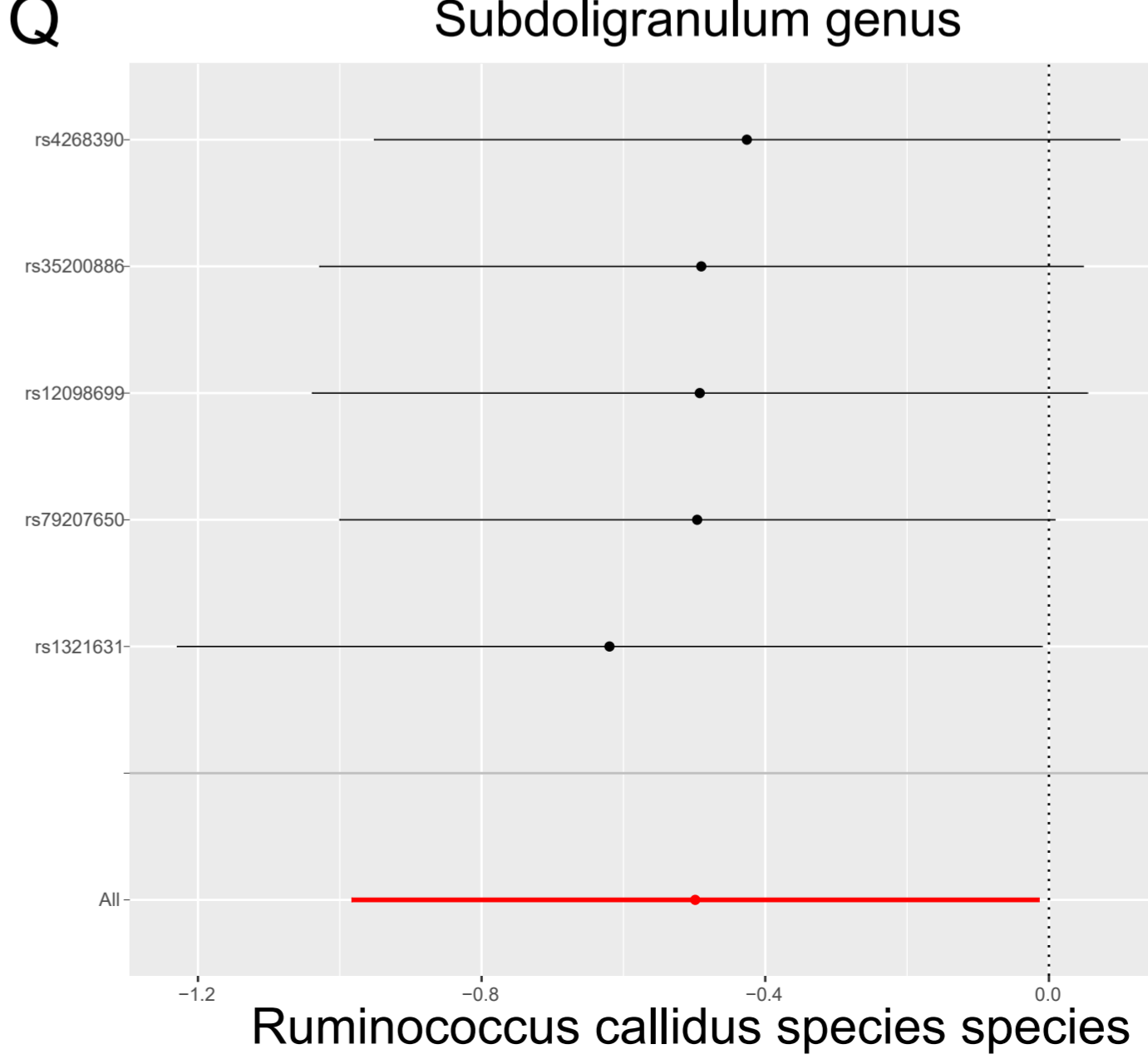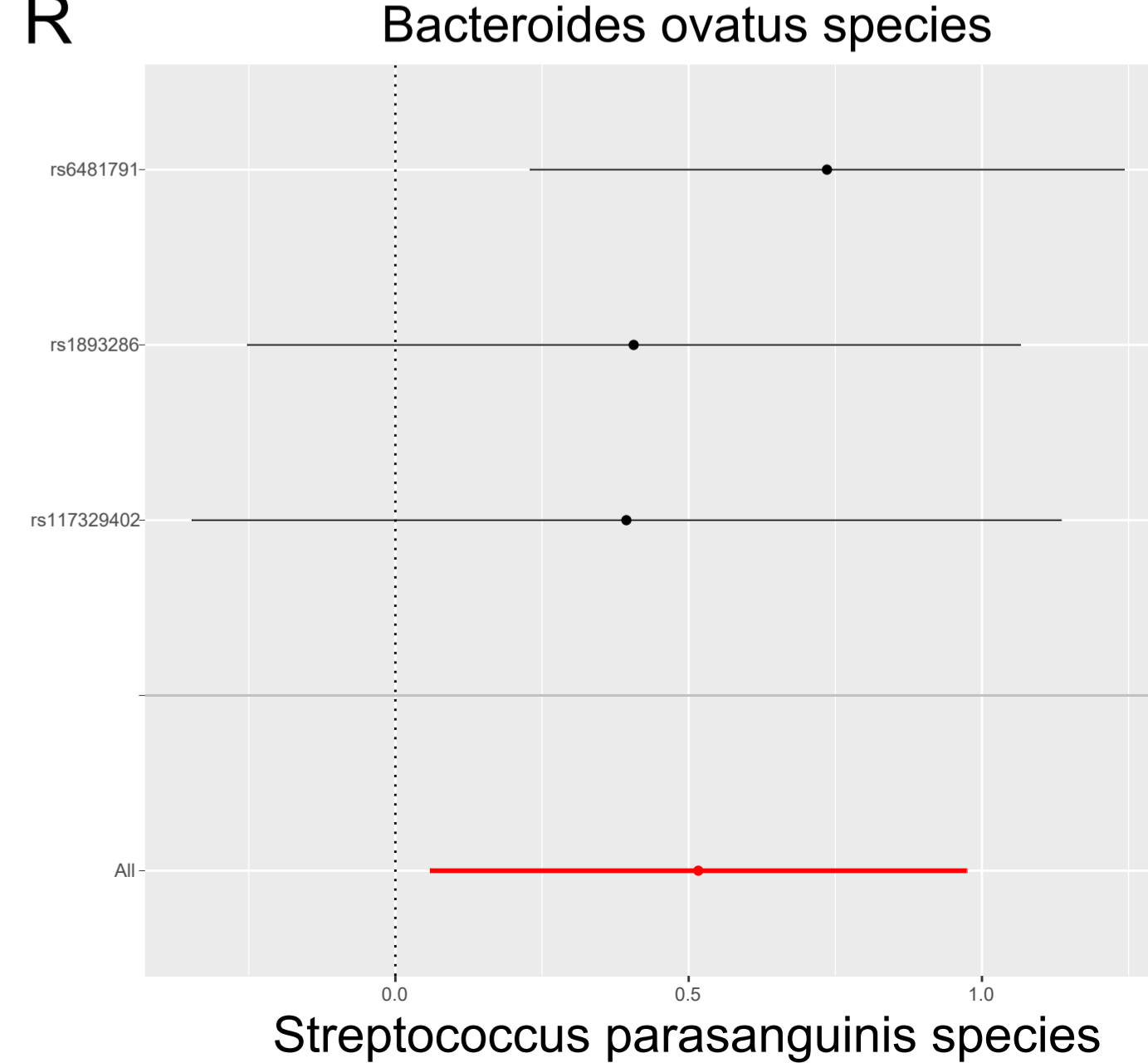

Supplement: Supplementary file 1 [file microorganisms-14-00667-s001.zip › Supplementary Figure 1.pdf]

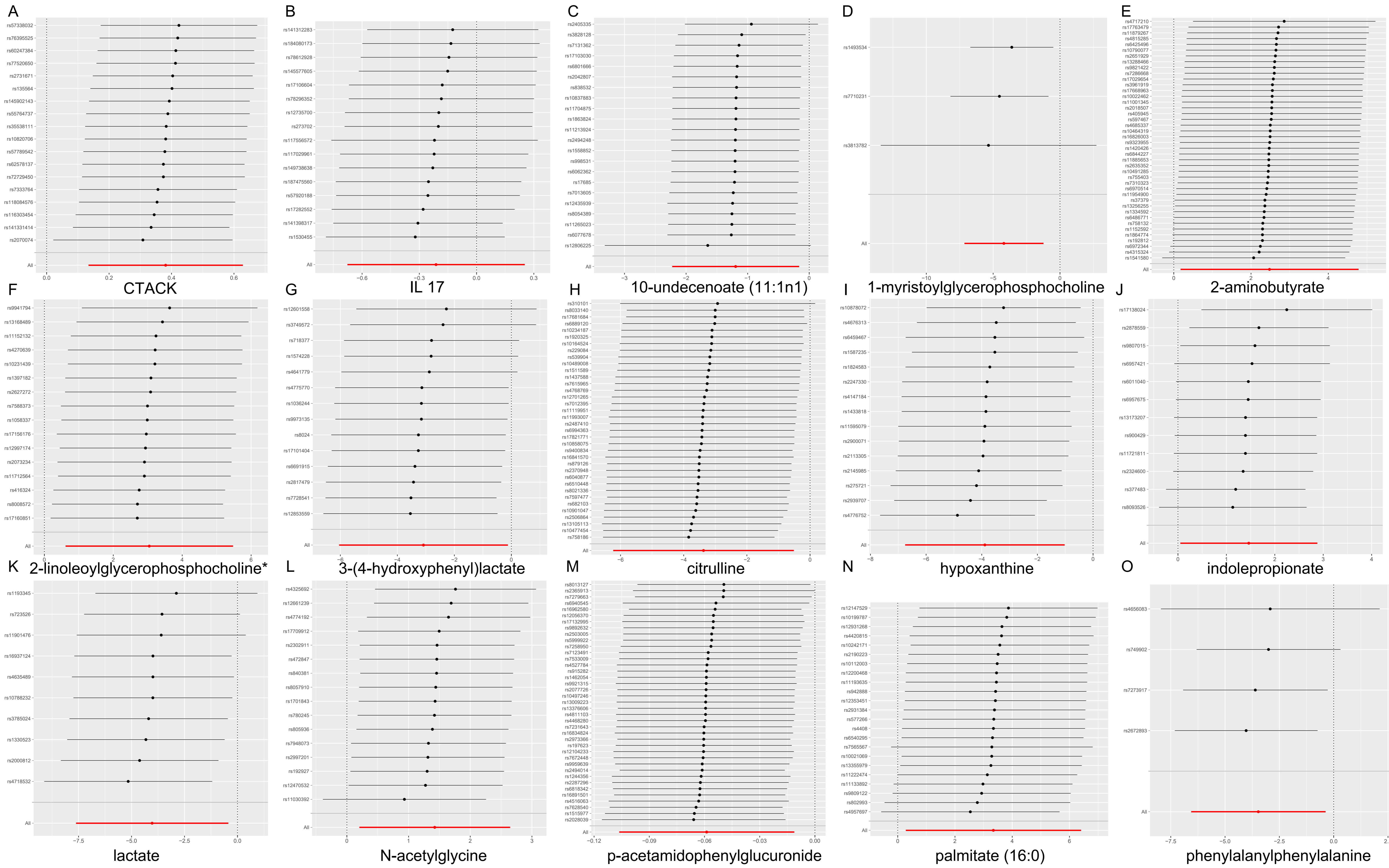

Supplement: Supplementary file 1 [file microorganisms-14-00667-s001.zip › Supplementary Figure 2.pdf]

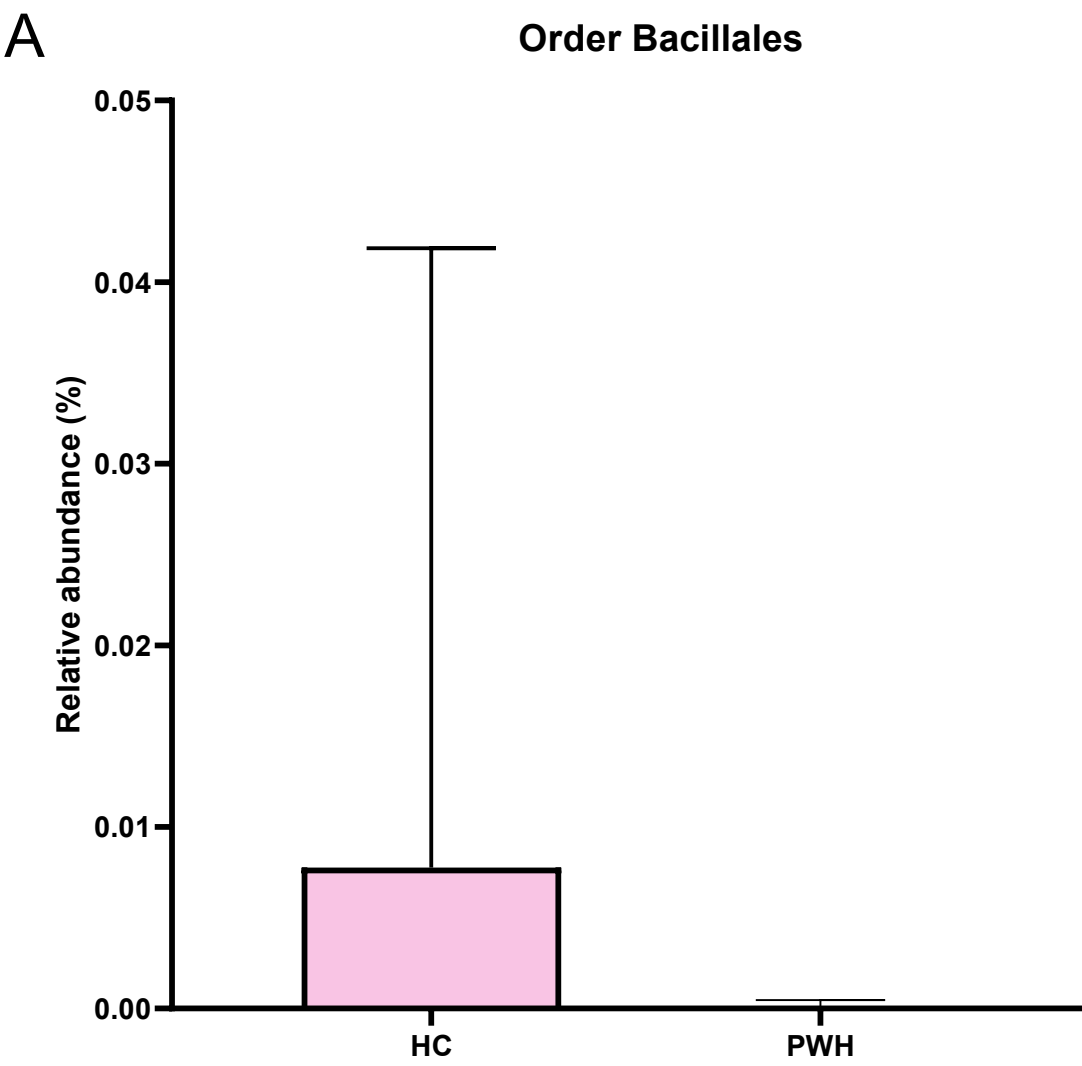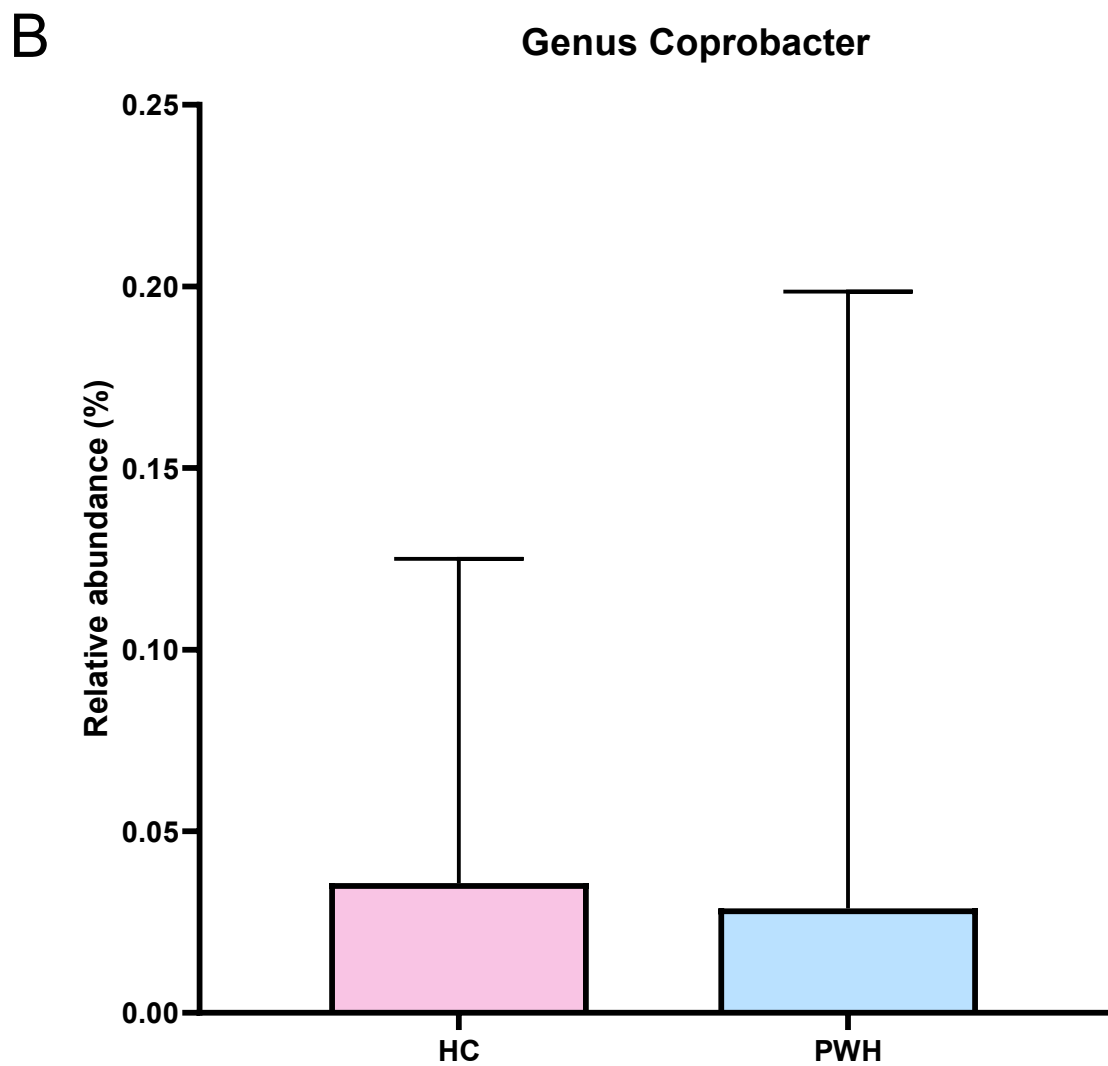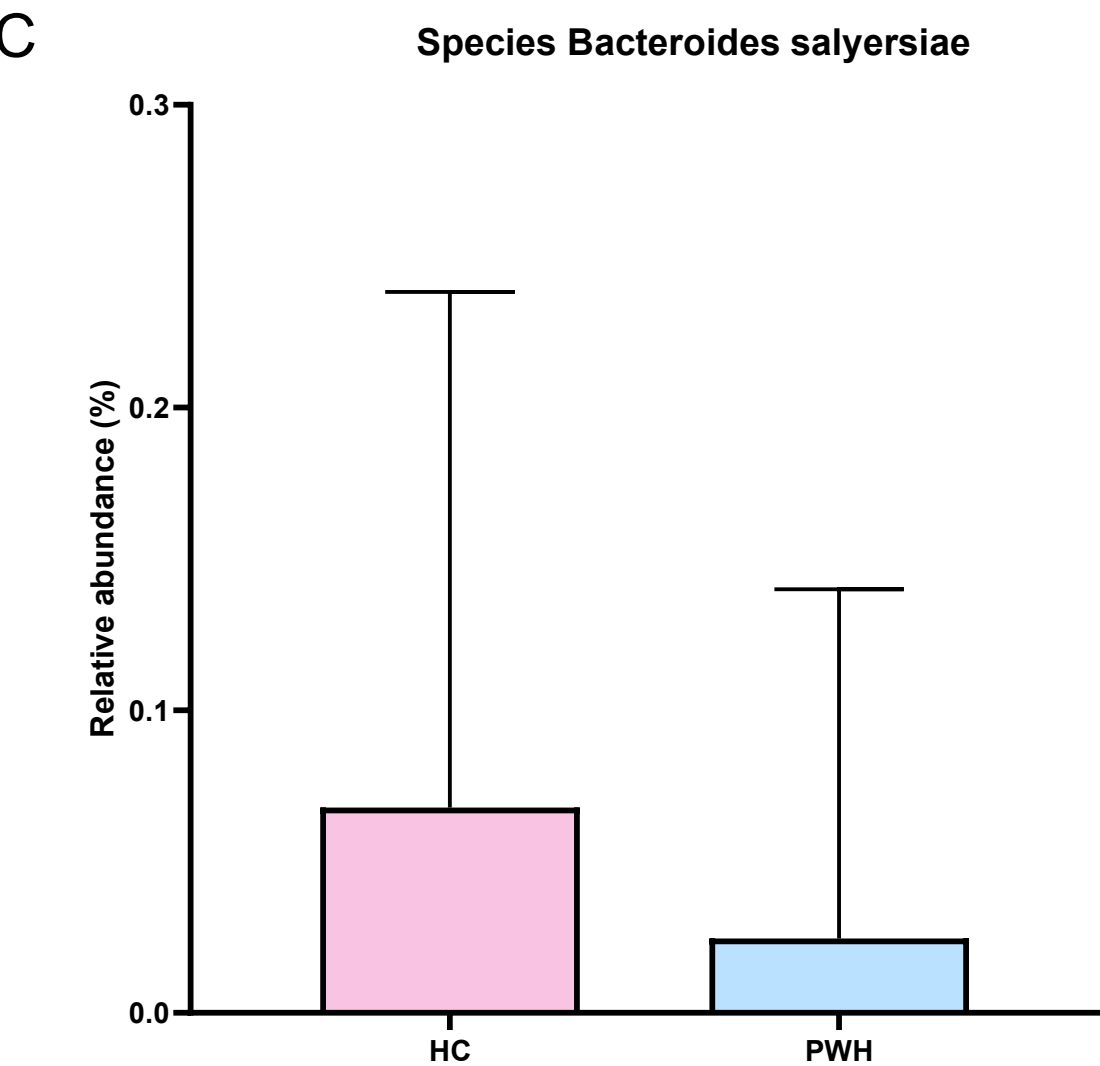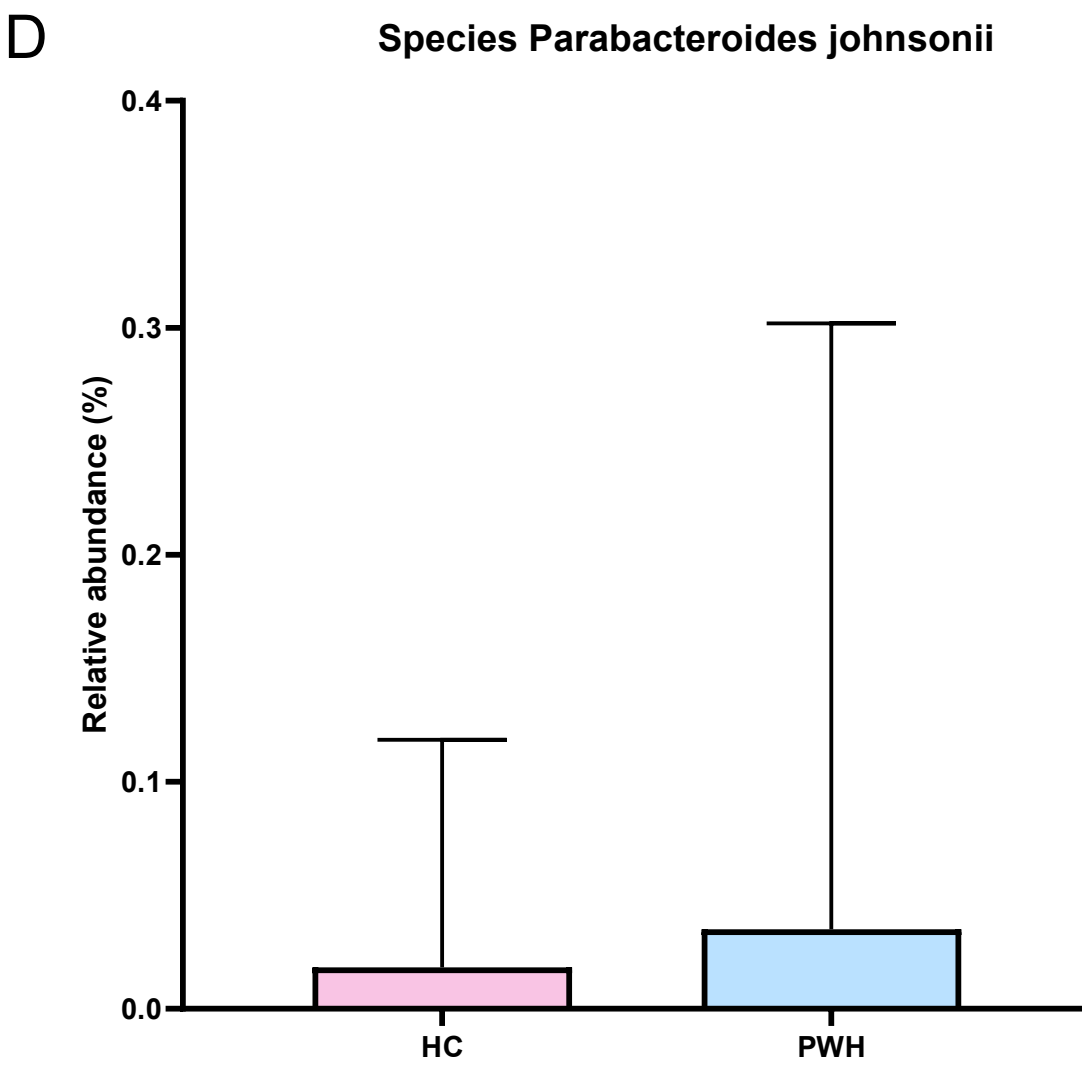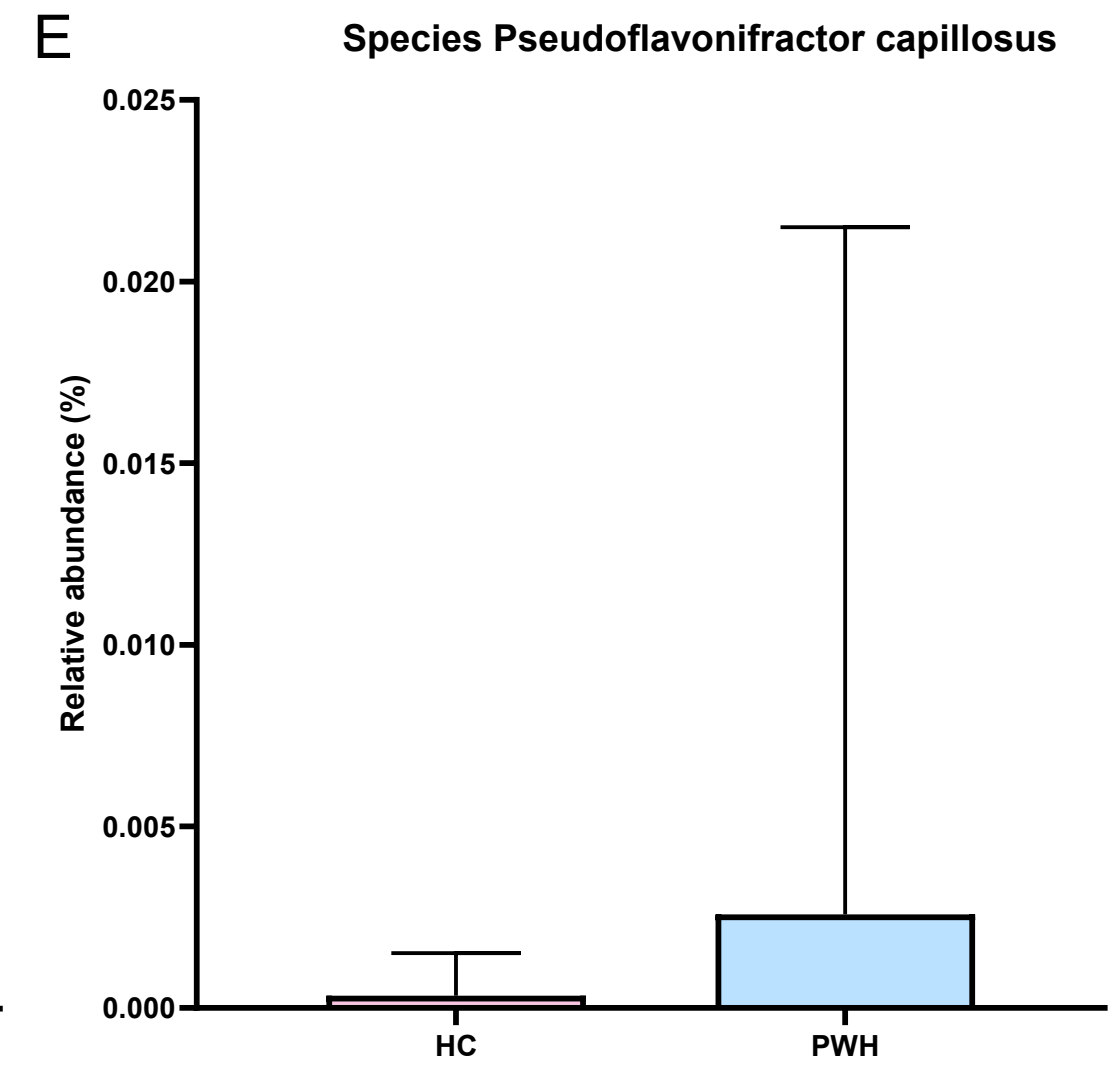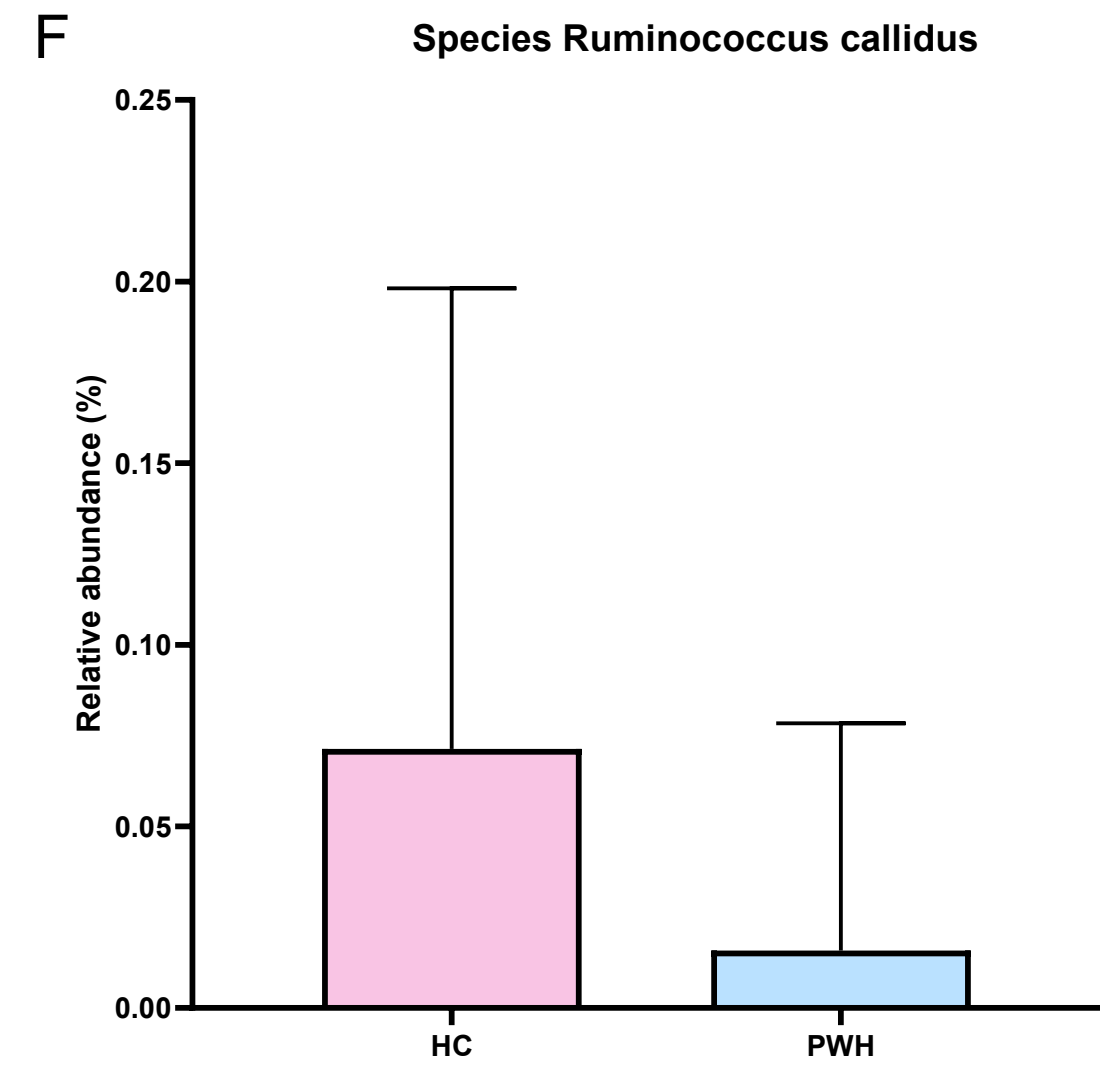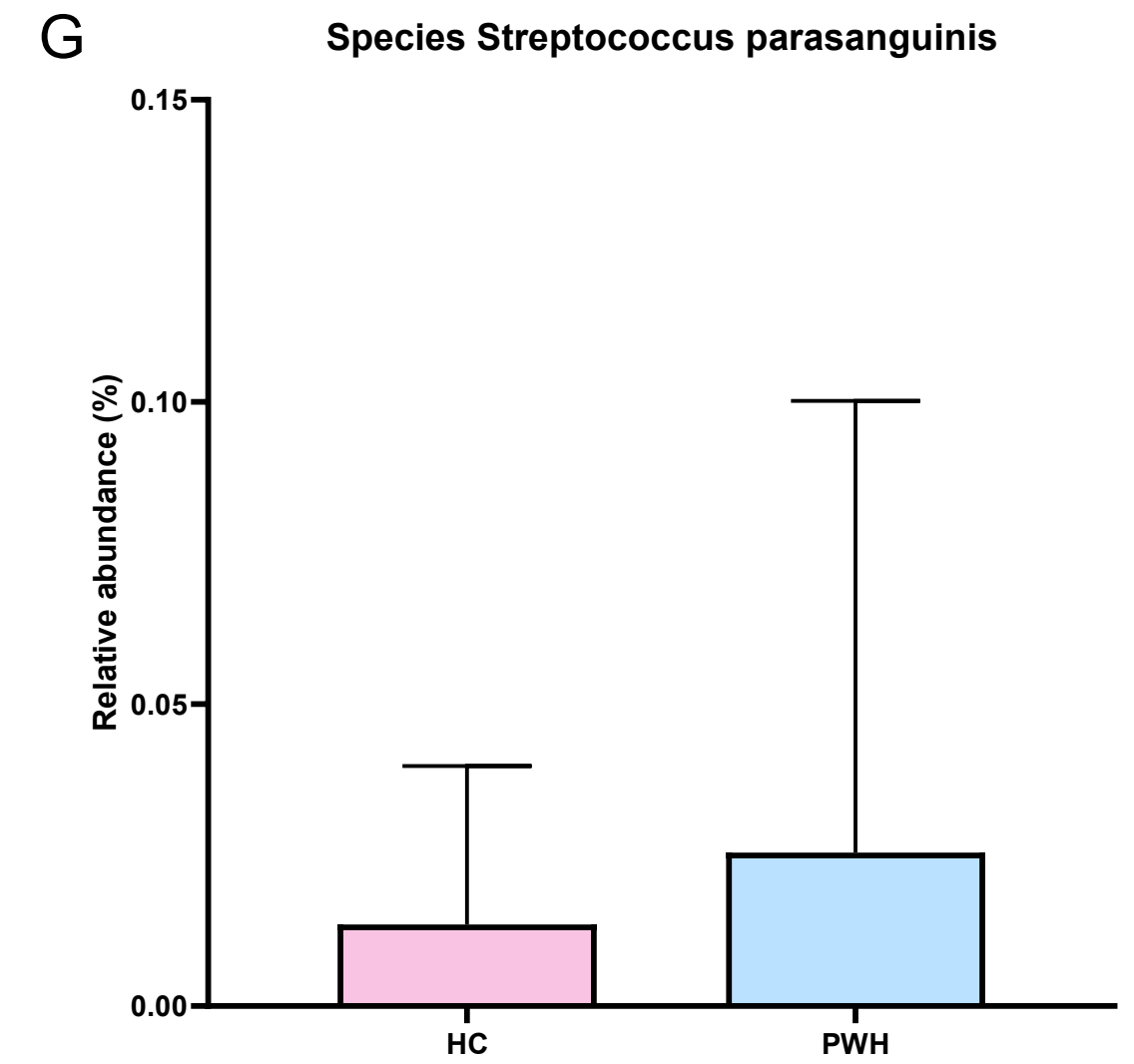

Supplement: Supplementary file 1 [file microorganisms-14-00667-s001.zip › Supplementary Figure 3.pdf]
